# Continuity and change in lithic techno-economy of the early Acheulian on the Ethiopian highland: A case study from locality MW2; the Melka Wakena site-complex

**DOI:** 10.1371/journal.pone.0277029

**Published:** 2022-12-07

**Authors:** Tegenu Gossa, Erella Hovers

**Affiliations:** 1 Human Evolution Research Center (HERC), The University of California at Berkeley, Berkeley, CA, United States of America; 2 Institute of Archaeology, The Hebrew University of Jerusalem, Jerusalem, Israel; 3 Department of History and Heritage Management, Arba Minch University, Arba Minch, Ethiopia; 4 Affiliate Researcher, Institute of Human Origins, Arizona State University, Tempe, AZ, United States of America; Griffith University, AUSTRALIA

## Abstract

Recent research has made great strides clarifying the chronology, temporal span, and geographic and technological patterning of the Acheulian in eastern Africa. However, highland occurrences of the Acheulian remain under-represented and their relationship to cultural dynamics in the Rift are still poorly understood. Recently, a stratified sequence of four archaeological layers, recording Acheulian occupations dated between ~1.6 Ma and ~1.3 Ma, has been discovered in locality MW2 of the Melka Wakena site-complex (south-central Ethiopian highlands). This database enabled a systematic exploration of the question of tempo and mode of technological changes at a local sequence, allowing, for the first time, comparison with other highland sites as well as in the Rift. The detailed techno-economic study presented in this study shows that the early Acheulian at the locality was characterized by the co-existence of lithic reduction sequences for small debitage and for flake-based Large Cutting Tool production. In the early, ~1.6 Ma assemblage, a strategy of variable raw material exploitation and technological emphasis on small debitage were coupled with production of few crude bifacial elements. These shifted at ~1.4 Ma towards a preferential and intensive exploitation of a highly knappable glassy ignimbrite and emphasis on Large Cutting Tool production, including higher investment in their techno-morphological aspects. The MW2 sequence tracks lithic technological trends observed in the Rift, with only a short time lag. Diachronic changes in the raw material economy and land use patterns may have occurred at MW2 earlier than previously reported for the Acheulian on the highlands. The behavioral dynamics gleaned from the early Acheulian assemblages at MW2 are important for our understanding of the diachronic changes in the abilities of Acheulian hominins to exploit the diverse geographic and ecological habitats of eastern Africa and beyond.

## 1. Introduction

Favorable preservation conditions and abundance of datable geological horizons have made the East African Rift System (EARS) a primary focus of paleoanthropological research over the last century, leading to major discoveries in both biological and cultural aspects of hominin evolution (see [1] for overview and references). In contrast, few early paleoanthropological sites/site-complexes have been reported from high elevation Rift shoulder contexts of Ethiopia and Kenya (~2000 m above mean sea level; hereafter highlands). Currently, only three site-complexes (Gadeb, Melka Kunture in Ethiopia and Kilombe in Kenya) have been subjected to long-term research programs including dating efforts and studies of lithic, faunal and hominin skeletal remains (e.g., [2–17]). Following from this history of research, current models of biological evolution, processes of demic expansions, and cultural changes in Early Pleistocene Africa do not address whole ecological and geographic range of early hominin behavioral adaptations. The highlands paleoanthropological record, while limited in scope, underlines the need to expand paleoanthropological research to the out-of-EARS areas.

**Fig 1:**
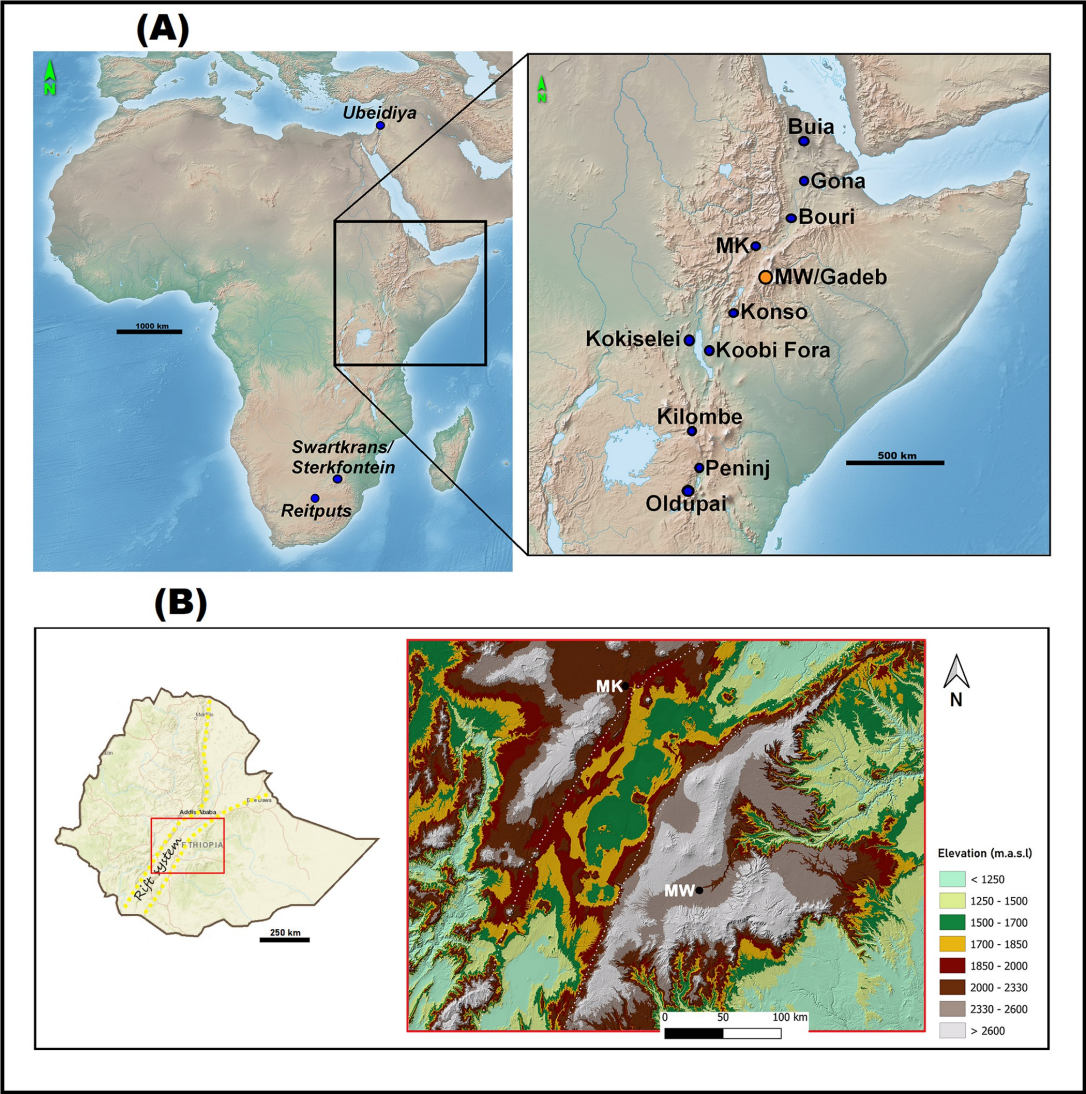
A) Location map of early Acheulian sites in Africa. Inset shows eastern African sites mentioned in the text. Relief map from Natural Earth (public domain): http://www.naturalearthdata.com/ B) Relief map showing the terrain around the Melka Wakena site-complex (referred to as **MW** in the map). The boundaries of the Main Ethiopian Rift are marked by white dotted lines. **MK** = Melka Kunture site-complex. DEM from USGS National Map Viewer (public domain): http://viewer.nationalmap.gov/viewer/. The map was created by authors in QGIS.

Paleoanthropological sites within the EARS document the earliest Oldowan occurrences at 2.6 Ma ([18], and references therein). The EARS is also the geographic origin of the Acheulian technocomplex, with its initial appearance at Kokiselei (KS4, [19]) and Konso (KGA6-A1, [20]) at ~1.75 Ma years and in Oldupai Gorge (FLK-West, [21]) at ~1.7 Ma, as well as the source region of the slightly later dispersals of Acheulian-bearing *Homo erectus* out of Africa [22–27]. On the highlands, the earliest records of Acheulian hominin activities are from locality Garba IVD at Melka Kunture and the recently-reported locality MW2 in the Melka Wakena (MW) site-complex, both dated to ~1.6 Ma ([1, 17], and references therein). (Fig 1). (An older date based on magentostratigraphy, which places this locality at ~1.9 Ma [28], is inconsistent with the published radiometric dating [9]).

### 1.1 The early Acheulian technocomplex

The emergence of Acheulian technology at ~1.75 Ma broadly overlaps with the appearance of *Homo ergaster/erectus* [29, 30], believed to have marked a fundamental transformation in the hominin lineage of physical traits [31–34], ecological flexibility [33, 35], and complex social behavior and organization [36, 37]. First recognized in southern Africa in the 1920s and the Oldupai Gorge in the early 1930s [38, 39], the Acheulian was characterized typologically by the incorporation of large bifacial tools (i.e., handaxes) into core-and-flake assemblages of the preceding Oldowan technocomplex, in gradually increasing frequencies [40, 41]. Isaac [42] considered the production of large flakes from giant cores, for the purpose of using them as blanks for the manufacture of Large Cutting Tools (LCTs), as a technological hallmark of the Acheulian.

Currently, the early Acheulian technocomplex is characterized by the coexistence of two distinct lithic reduction approaches—one for the production of small debitage, with various techno-typological elements continuing from the Oldowan technocomplex, and another for the newly established manufacture of LCTs [8, 18, 30, 43–46]. Both the small flake and LCT production systems are said to feature variability of lithic techno-economies, resulting from raw material properties and preferences (e.g., size and lithology), increasing diversity of knapping methods within each flaking system, and increased complexity of landscape use strategies [8, 20, 21, 44, 47–51].

Despite the growing focus on the study of the whole assemblages (as opposed to emphasis on specific tool types) and on inter-assemblage variability [18], the Acheulian is still characterized by bifacially shaped handaxes as its emblematic tool. The functions and shape characteristics of handaxes are variably explained, ranging from their efficiency in mega-fauna butchery [52–57] and woodworking [58–60] to their suitability for processing vegetal underground storage organs [61], arguably combined with their ergonomic benefits for grasping by hand [62–64]. Other researchers, however, claim that their shape is the unintended outcome of their use as cores for flake production (e.g., [65–67], or of continuous reduction process (e.g., [68, 69]). Yet other researchers emphasized the socio-cultural context and meaning of these tools: the societal investment needed to produce skilled knappers [70, 71], the complex social organization implicated for LCTs production and use [37, 72–74]; their symbolic and aesthetic function/values [75–80]. Some researchers questioned whether these tools were the product of exclusively cultural transmission (e.g., [81–84]), while others suggested that they were the products of more or less specific genetically-determined behavior (e.g., [85]; but see [86]), or were easy to reinvent given a certain combination of cognitive level and technological knowledge (e.g., [87]).

The methodological and conceptual limitations of the over-dependence on bifacial tools in Acheulian research have long been realized [41, 88–90]. Once a bi-modal classificatory scheme of knapping processes into the debitage and façonnage operational stages was incorporated in current research on the Acheulian [91], significant strides have been made in the conception of the Acheulian technological system and understanding the lifeways of hominins that utilized this technology [8, 21, 34, 44, 47–51, 92–98]. In recent years, researchers have realized the importance of LCT production processes (rather than the shape itself) as a means of deciphering the full spectrum of the technological knowledge and its underlying behaviors and cognitive abilities (e.g., [13, 21, 47, 99]).

While the available evidence speaks to the tempo of the emergence of the Acheulian at 1.75 Ma, it is less clear about its mode of change from the Oldowan and about its diachronic development. Leakey’s [40] view of a gradual shift from the Oldowan through phases of a transitional “Developed Oldowan”, despite some early critiques (e.g., [100–102], has become the standard viewpoint and applied in numerous instances well into the early years of the 21^st^ century (e.g., Chesowanja [103]; Melka Kunture [104], Gadeb (Gadeb 2B, 2C, 2E, and 8F; [2, 105, 106], and Koobi Fora [107]). Although renewed work has considerably undermined this view [8, 30, 43, 44, 93, 108–110], there are disagreements about the causes and the mode of transition to a full-fledged Acheulian technology (e.g., [18]). Thus, researchers debate whether this was a gradual process where Oldowan components continued to exist side by side with novel Acheulian characteristics until they were eventually discarded [8, 111], as opposed to an abrupt behavioral response to changing ecological conditions (increasing aridity in the context of climate pulses) by a newly emerged hominin species (*H*. *erectus*) (e.g., [30, 43, 112, 113]). Notably, the emblematic bifacially shaped LCTs are extremely rare or absent from some key early assemblages that post-date the earliest Acheulian (e.g., Peninj-ST site-complex, [114]; Gombore Iγ and Iδ, [17, 48]. This further confounds the question of the mode of cultural change.

With an age range of ~1.62 Ma to ~1.34 Ma [1], the stratified archaeological occupations at locality MW2 fall within the early stages of the Acheulian and provide information about the question of the tempo and diachronic changes of lithic techno-economic behaviors along the local sequence. Furthermore, the site’s topographic location allows comparisons to other highland localities, providing for the first time insights into the variability of lithic systems in this physiographic context, as well as assemblages in the Rift.

### 1.2. Site settings and the context of MW2 lithic assemblages

**Fig 2:**
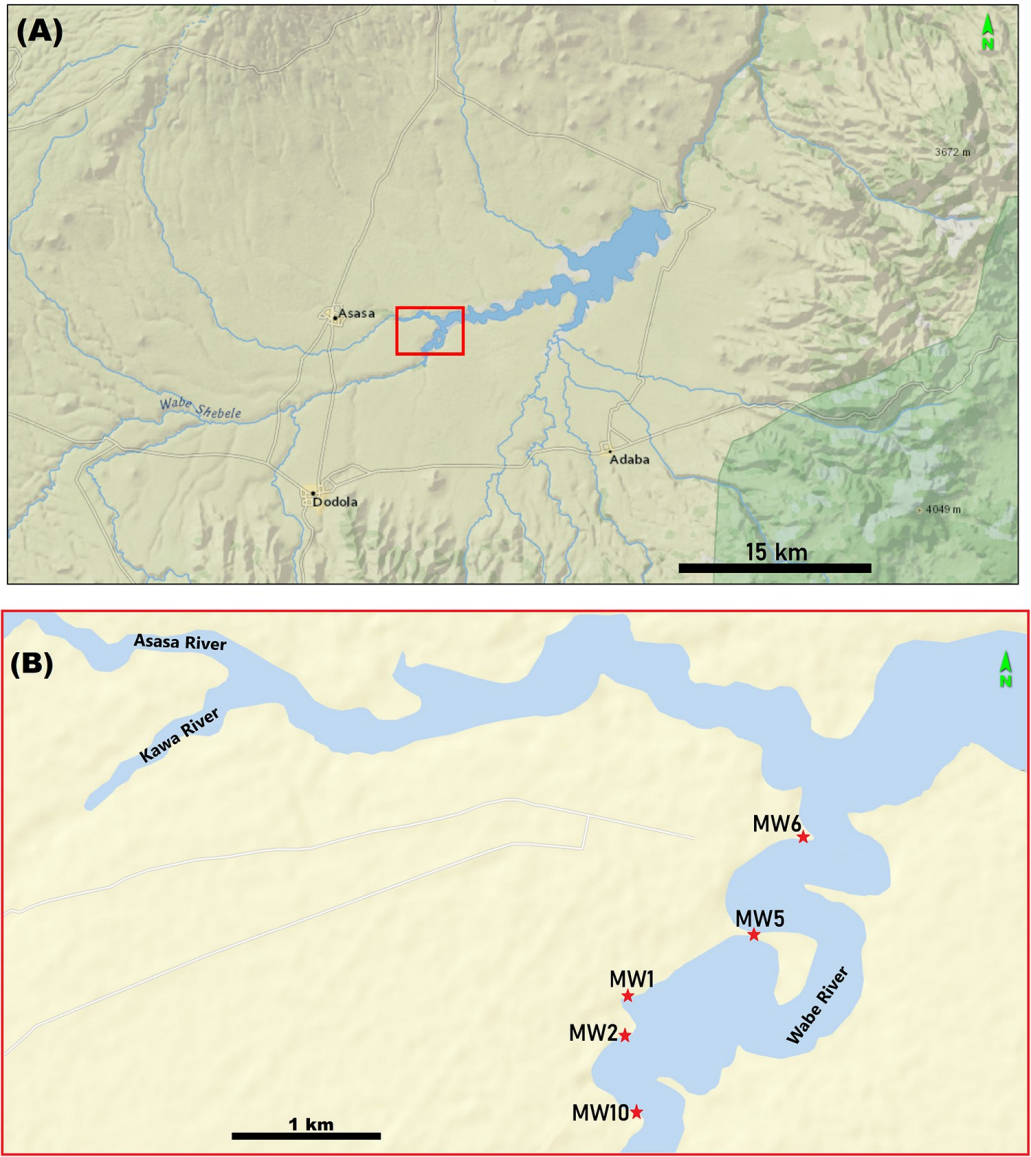
A) Terrain map showing the general view of the Gadeb Plain B) Location of some of the archaeological localities along the meandering course of the upper reaches of Wabe River. Terrain map from USGS National Map Viewer (public domain): http://viewer.nationalmap.gov/viewer/.

The MW site-complex is situated on the eastern shoulder of the Main Ethiopian Rift, at 2300–2350 m above mean sea level at the headwater of the Wabe Shebele drainage system (Fig 1A), in sediments associated with the early to early Middle Pleistocene Dino Formation. Ten localities were identified thus far in the site-complex (Fig 2). Based on stratigraphic and radiometric dating, the localities of Gadeb [2, 4, 105, 106], located ~8 km downstream of MW, were situated on the same paleo-flood plain (‘MW-Gadeb plain’). Since the Gadeb site is permanently inundated [8], more detailed stratigraphic and temporal relationship to MW cannot be established.

The Dino formation in the MW area consists of well-sorted and uniformly thick distal volcanic products that are associated with the Plio-Pleistocene activity of the large rift calderas. Rhyolitic lava flows, ash flows, pumice, pumaceous ash falls and welded tuffs originated from the nearby large silicic central volcanoes on the eastern rift shoulder (Fig 1B) [115]. The time span of hominin presence within the site-complex is ~1.62 Ma to ~0.69 Ma, based on ^40^Ar/^39^Ar dates obtained from nine tephra horizons within the site’s stratigraphic sequence (see [1] for details on all MW dates mentioned in the current paper). Geological, geochronological and sedimentological data ([1, 115], Resom A. [Unpublished]) suggest that during the time of the Early Pleistocene to the early Middle Pleistocene the MW-Gadeb Plain was a hydrologically active area, where the pyroclastic materials were intercalated with fluvial deposits derived from channels flowing off the Bale mountain-range into an extensive MW-Gadeb flood plain. The plain contained a dense network of meandering channels and streams as well as temporary ponds. The fluvial activity caused at times reworking, transport and redeposition of primary pyroclastics in channel fills [4, 115]. Stratigraphic and geochronological data imply higher channel energies in the later time periods with higher post-depositional consequences for the younger archaeological assemblages [1]. Faunal remains from the Early Pleistocene archaeological and paleontological strata indicate a mixed landscape of open grassland and forested areas in proximity to water bodies.

**Fig 3:**
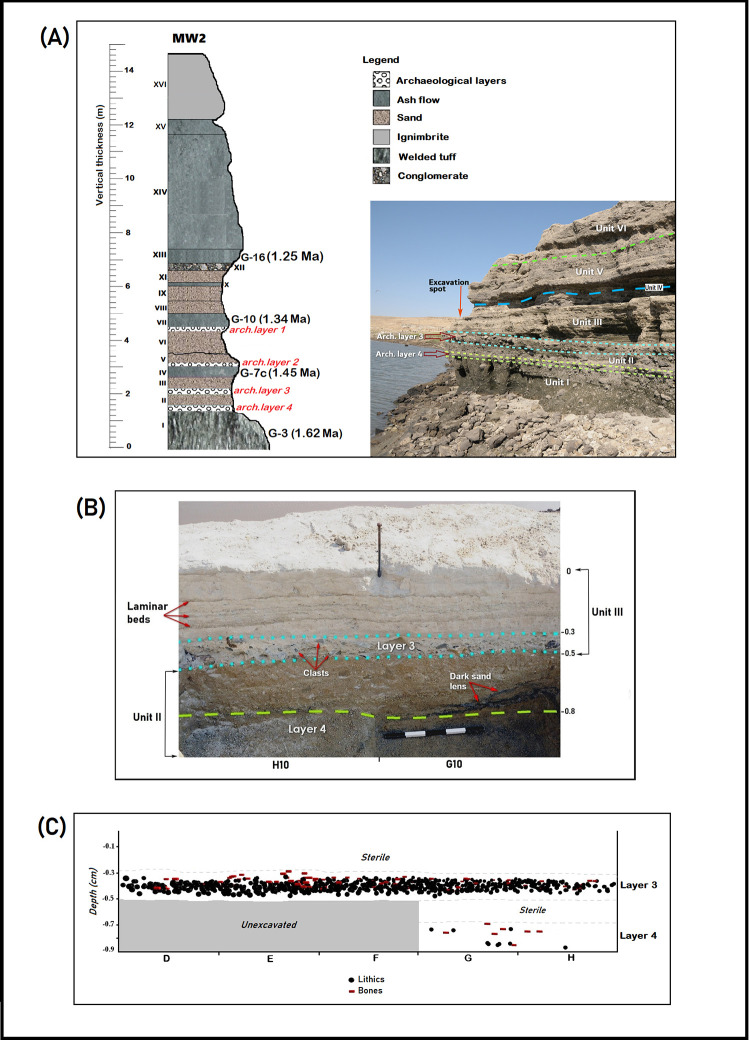
A) The stratigraphic sequence of MW2, showing the archaeological layers in relation to dated tephra. The photograph on the right shows the cliff face composed of the bottom part of the sequence (geological units I-VI) in which the archaeological layers are embedded. Note that the excavated area itself is not shown in this view. B) a partial view of the southern profile of the excavation (along squares G10 and H10), showing higher resolution details of the sedimentary make-up of geological units II and III and the stratigrpahic positions of MW2-L3 and MW2-L4 in relation to the sedimentary changes. C) Stratigraphic and lateral distributions of piece-plotted artifacts and bones in MW2-L3 and MW2-L4, projected onto a 2D-view.

MW2 is one of three localities tested to date by small-scale excavations of the Melka Wakena Paleoanthropological Project (MWPP) each of which contained stratified archaeological horizons. All the occupations were associated with overbank or fluvial activity [1]. The archaeological layers in the 14.6 m-long cliff-section of MW2 are embedded in a sequence of pyroclastics interbedded with fluvial and overbank deposits (Units I-VII; Fig 3A). ^40^Ar/^39^Ar dates place the deposition of archaeological layers L4 and L3 (hereafter MW2-L4 and MW2-L3) between 1.6225 ± 0.0039 Ma (Unit I, which is archaeologically sterile) and 1.4451 ± 0.0193 Ma (Unit IV). These layers are embedded in an upward fining sand sequence representing low-energy fluvial activity. Archaeological layers L1 and L2 (hereafter MW2-L2 and MW-L1) are older than 1.3414 ± 0.0041 Ma (Unit VII). MW2-L2 and MW2-L1 are associated with a loose, fine conglomerate layer (Unit V) and a loose, well-sorted coarse sand layer (Unit VI), respectively; representing a higher energy fluvial system than the underlying sequences embracing MW2-L4 and MW2-L3. (More details on the geology, paleo-landscape and localities at the site-complex can be found in [1, 115]).

### 1.3. Research questions

Lithic assemblages are archaeological proxies of early hominin decision-making regarding ways of making stone artifacts and how they are used and discarded across the paleo-landscape to help hominin physical and social survival in their varied ecological niches.

There are currently significant differences in the ecological conditions between the Rift Valley and the adjacent highlands, including the amount of rainfall and its seasonal distribution as well as levels of solar radiation. Moreover, the afro-montane habitat existed on the highlands at least from 1.8 Ma, suggesting a different habitats and biodiversity on the highlands compared to the Rift Valley from this time. Moreover, while many animal species are similar or identical in the two habitats, there are few that are confined to one or the other (as discussed and referenced in [1]).

It has long been assumed that human adaptations through mobility, raw material use and transport, and possibly the toolkits in these two environments differed to some degree. Three models have been formulated to explain the Acheulian connections between the Rift and the highlands, focusing on different scales of mobility as inferred from lithic assemblages. Clark and Kurashina [3] suggested cyclic, possibly seasonal, movements of Acheulian groups from the Rift to the highlands over long (>100 km) distances, based on sourcing of a scanty number of lithic artifacts. Alternatively, *Homo erectus’* occupations on the highlands were hypothesized to represent a large-scale expansion of the range of exploited habitats [106], with implications for artifact densities and assemblage compositions in different ecological contexts. A mode of larger-scale climate changes as the drivers of mobility patterns was suggested by Mussi et al. [116], who attributed the discontinuous occupation at Melka Kunture to cyclic climate deterioration and ameliorations. However, the paucity of Early Pleistocene highland occurrences has severely undermined researchers’ ability to gain insights into inter-assemblage lithic technological variability within highland early Acheulian occupations, their dynamics of change and continuity, and the environmental background to such dynamics. Such lacunae also hampered the ability to address the tempo and mode of technological changes within highland Acheulian assemblages and how such changes compare with those described for Rift Valley occurrences.

Data about the material cultural remains from the early Acheulian locality of MW2 lithic assemblages allow, for the first time, inter-assemblage comparisons that are based on comparable methodologies, thus helping to create the necessary database for broader regional comparisons. Specifically, we present a techno-economic study of the MW2 assemblages that elucidate the technological choices of hominins in using lithic raw material and adopting ways of making stone tools. We focus on lithic technology including raw material selection patterns, blank preparation, blank selection for secondary modification and discard patterns, taking into account taphonomic and sample size constraints. When placed against the geochronological framework of the locality, the results allow tracking of diachronic trends of continuity and change. We are then able to ask whether early Acheulian lithic technology at the locality changed at a single pace as a discrete technological package, or whether changes attributed to specific stages of the operational schemes emerged piecemeal, emerging as a changed phase of early Acheulian technology.

## 2. Materials and methods

The MWPP conducted archaeological work at locality MW2 during the month of February 2016, with a permit obtained from the Ethiopian Authority for Conservation of Heritages (EACH). Subsquent analyses and curatorial work were carried out in the EACH facility in Addis Ababa.

### 2.1. Excavation and sampling procedures

We identified four discrete archaeological horizons at MW2 (Fig 3A). The two younger layers, MW2-L1 and MW2-L2, are situated in the fluvial context of MW2-Unit V and VI (Fig 3A). The two horizons were too heavily disturbed by quarrying acitivities, due to which artifacts from both layers had been displaced from their *in situ* locations. As a result, systematic excavations of the *in situ* occurrences of MW2-L1 and MW2-L2 were not possible at the time. Still, since the *in situ* horizons are stratigraphically as well as topographically higher than the *in situ* MW2-L3 and MW2-L4 occurrences, the artifacts from the slope were assigned to ‘MW2–L1&L2’ without further distinction. Based on the site’s sequence, these artifacts are attributed to a well designated temporal range (i.e., ~1.45- ~1.34 Ma; see section 1.2.). We collected all visible items of MW2-L1&L2 from an area of 20 m^2^. The fresh condition of the scattered artifacts is consistent with information from the local quarry workers, indicating recent exposure onto the surface over the last 2–3 years prior to our field operations.

The MW2-L1&L2 assemblage was used in comparative diachronic analyses of selected types (larger items that were collected fully from the designated surface area), but was not used in analyses focusing on inter-assemblage structure, to avoid false patterns due to collection bias against certain artifact classes (e.g., regular flakes, small flakes, flake fragments, and debris).

In MW2-L4 and MW2-L3, *in situ* finds were collected from a virtual site-specific 1 x 1 m grid system. Three-dimensional coordinates were obtained for each visible find encountered during excavation, using a Sokkia 630 total station. All the excavated sediments were dry-sieved in a 5mm mesh according to grid and vertical spits and all recovered lithic artifacts and clasts were included in the current analysis.

**Fig 4:**
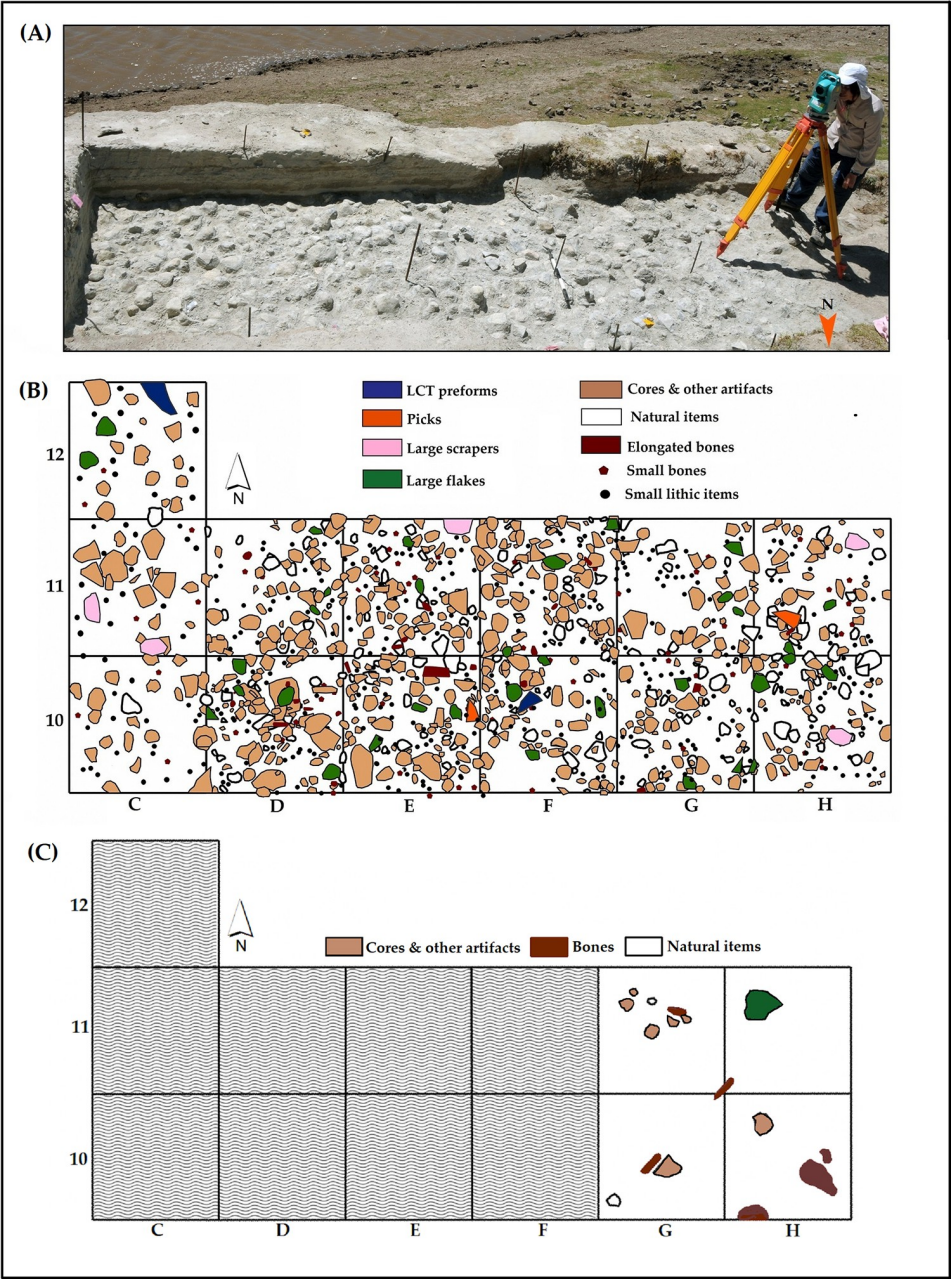
A) MW2-L3 during excavation in 2016 (view to the south). Georeferenced distribution map of MW2-L3 (B) and MW2-L4 (C) lithic and fauna elements.

MW2-L3 was excavated over an area of 13m^2^. A total of 6,738 lithic clasts (of which 6,064 were identified as artifacts; see Table 1) and 87 highly fragmented faunal remains were retrieved from the excavated area (Fig 4A and 4B; Table 1). Artifacts were clustered in a relatively thin horizon (~20 cm-thick) at the bottom of MW2-Unit III (Fig 3B and 3C). With an artifact density of 2,332 artifacts per 1m^3^, MW2-L3 contains the largest concentration of lithic artifacts among the localities tested so far in the site-complex. (Note that this density value differs from previous published ones [see [1], their Table 3] following re-assessement of the artifactual status of some of the lithic clasts.)

**Table 1:** Composition of the lithic assemblages of MW2 occupation layers.

Clast category	MW2-L4		MW2-L3		MW2-L1&L2	
	*n*	*%*	*n*	*%*	*n*	*%*
Cores^a^	2	1.92	259	4.27	40	20.0
Debitage^a^	101	97.11	5696	93.93	114	57.0
LCTs^a^	-	-	12	0.19	36	18.0
Retouched tools^a^	-	-	4	0.06	1	0.5
Hammerstones^a^	1	0.96	46	0.76	7	3.5
Modified items^a^	-	-	47	0.77	2	1.0
**Total artifacts**	104	100.0	6064	100.0	200	100.0
% artifacts^b^	100.0		90.0		100.0	
Natural items^b^	-	-	101	1.5	-	-
Indeterminates^b^	-	-	573	8.5	-	-
**Total assemblage**	104		6738		200	

^a^Percentage in total artifacts.

^b^Percentage in total assemblage (i.e., including natural and indeterminate pieces). **LCTs** = large cutting tools.

Note: Discrepancies between the inventory of MW2-L 3 reported here and the one in Hovers et al. [1] are due to an updated reanalysis of the assemblage, leading to the current quantitative breakdown.

MW2-L4 was excavated over only 4 m^2^ (Figs 3C and 4C). It contains mainly sparsely scattered bones in a coarse, loose sand at the bottom 20 cm of MW2-Unit II. The bones are associated with few lithic artifacts. Anthropogenic percussion marks on hippo and bovid bones indicate activities related to the exploitation of faunal resources (see [1], their Fig 10).

### 2.2. Raw material survey and sampling

We conducted systematic pedestrian surveys covering distances up to 20 km in the area of the MW site-complex during 2016 and 2017 field seasons. Columnar logs were described across the area of the MW site-complex ([115], Resom A. [Unpublished]), incorporating information about the stratigraphic relationship between alluvial and pyroclastic deposits on the one hand and flows of volcanic rock that were hypothesized to have been potential sources of raw material for the lithic assemblages, based on post-excavation naked-eye analyses. The exposed flows were sampled for petrographic thin section analysis (*n* = 9) (S1 Text) that was carried out in the School of Earth Sciences of the Addis Ababa University. Three ignimbrite samples were also analyzed for major and trace geochemistry [115].

### 2.3. Analysis

The ‘*chaîne opératoire*’ approach was used to study the lithic technology of the MW2 assemblages. This approach attempts to elucidate aspects of the knappers’ cognitive and technical abilities, as well as the socio-economic organization of activities, from the implementation of technological processes [117–119]. Following from that, technological decisions of prehistoric hunter-gatherers can be better understood [118, 120–122].

A detailed attribute analysis was used to document a set of attributes of cores, LCTs, hammerstones, and flakes. This method enables a quantified description of the variation of metric, physical, and technological attributes and thus allows for formal testing (through descriptive statistics, correlations and simple linear models) of the relationship between artifact traits and their impact on the processes of manufacture. The quantified information can be translated into sequential models of the technological procedures, providing a framework for reconstructing knapping behaviors. Technological practices applied by knappers can be inferred from the patterning of the quantified variation of artifacts. Since lithic production is a reductive process that constitutes a sequence of actions upon matter, the quantified information can be ‘reverse engineered’ to model the technological procedures ([122] and references therein).

The selection of attributes in the current study is based on published studies that applied the same conceptual analytical framework to similar materials [22, 123]. Additional variables were constructed in response to assemblage-specific characteristics when they were observed during data collection. Documenting raw material, lithic taphonomy and metric variables and some of the technological variables (number of scars, cortex presence) was standardized for all artifact classes in the assemblage. For LCTs and cores, additional variables were documented in order to capture the variability of specific properties for these artifact categories.

Provenience variables document the 3D location of an artifact in the virtual site grid and enable analysis of spatial patterning, enabling reconstruction of the vertical and horizontal spatial distributions of finds. Lithic taphonomy–i.e., processes that affected the artifact after discard–is described through variables describing surface alterations (freshness of the artifact surface, exfoliation, adhering encustation, patination, post-depositional breakage) which can result from non-anthropic agents (e.g., water action) as well as unintentional (e.g., trampling) or purposeful (e.g., recycling) hominin behavior at the site. The combination of provenance and lithic taphonomic variables informs about the extent of artifact displacement related to presence of syn- and post-depositional site formation processes, their spatial variation across the excavated areas, and whether such variation is differential (e.g., according to raw materials, artifact size, mass, and shapes). Taken together, these observations help differentiate between human vs. non-human effects and form the basis for further inferences about hominin use of the lithic resources.

Each artifact was assiged to a raw material category by the naked eye. The identifications were compared visually to the geological samples that have been studied through petrogrpahy and geochemical analyes (see S1 Text). When quantified, the distribution of raw materials reveals preferences (or lack thereof) for certain raw material; when combined with typo-technological variables, this shows whether there are non-random links between certain raw materials and artifact morphotypes.

Identifying the procedures employed during core reduction (expressed by both core and flake characteristics) and in a later shaping stage (if the blanks are further modified) is crucial in the reconstruction of the lithic production processes, which in turn is useful in understanding the nature of technological flexibility (or lack thereof) and raw material economy of the knappers. Attributes observed on cores, flakes and modified items are used separately and in combination to obtain this goal. The term ‘modified items’ is used in this work in reference to cobbles/pebbles that manifest surface modifications. In this category are included modifications that resemble flake removal scars but cannot be confidently categorized as either cores or percussive materials. ‘Indeterminates’ are items with physical properties too ambiguous to categorise them as either artifacts or natural items.

The early Acheulian in Africa is usually known for the co-existence of two reduction sequences (i.e., small to medium-sized flakes and LCTs reduction sequences; [8, 18, 43, 44]). In this study, size measurements for cores and flakes followed methods devised by Goren-Inbar and Saragusti [124] and Sharon [125] (see S4 Fig). We follow previous authors [20, 43, 125] in setting the threshold value between small-to-medium and large flakes at ≥10 cm. Therefore, threshold values for separating cores into one of the two reduction sequences are determined based on the largest dimension (either length or width) of the dominant flake scar on the core surface. All measurements were held using Mitutoyo digital caliper 500–182 (resolution 0.01 mm).

The study of the reduction sequence of LCTs followed the bi-modal scheme [91; and see above], namely, the ‘debitage’ and ‘façonnage’ operational sequences, employed by some Acheulian researchers (e.g., [46, 49, 94]). For LCTs, the flaking orientation of flake blanks provides direct clues as to the specific technical procedures employed to obtain the blanks from giant/boulder cores and how pre-planned they may have been. This in turn would have implicaitons for the depth of planning involved in the production of LCTs. In the MW2 assemblages, five types of LCTs were reognized: Crude LCTs, when bifacial shaping led to an overall massive and rough aspect; picks, handaxes, cleavers and large scrapers. At least three types of flakes were encountered: side-struck, special side-struck and end-struck flakes ([98] and references in there; see S1 Text for definition and description; S1 Fig).

Measurements for the LCTs followed a method initially developed by Roe [126], including measurements of length of cutting edge, circumference, and weight [123–125] (see S4 Fig). The number of flake removal scars on each face of bifacial tools is used as a proxy for the intensity of bifacial shaping (façonnage; [125, 127]). In addition, indices of relative thickness and elongation were calculated for LCTs from the primary measurements.These two key variables speak to the shape aspects and flaking intensity of the bifacial tools as well as their functional viability [80, 128–131].

The technological characterization of core reduction methods was based on the classification used by de la Torre [8] in the study of the Gadeb assemblages. This scheme identifies three technological characteristics (unifacial vs. bifacial exploitation; core rotation; organization of the knapping surfaces) expressed archaeologically by the number of knapped surfaces, their geometric relationship to one another, and the geometric relationship between flake removals on each surface (see S1 Text and S2 Fig for details).

The quantified data were analyzed using the software PAST v. 3.21 and Microsoft Excel 2016. The outputs were used to quantitatively and graphically describe the patterns of intra-and inter-assemblage variability of MW2 assemblages and to test the strength of the patterns observed. Statistical tests were employed to evaluate the relationship between some variables (such as the sizes of various raw materials exploited as cores, hammerstones, and unmodified natural items) and their potential influence of the decision-making processes by knappers. As distributions deviated from normality, we employed the non-parametric tests of Kruskall-Wallis (K-W) and Mann-Whitney (M-W) to test for differences between artifact categories. For all tests, significance is α = 0.05.

## 3. Results

### 3.1. Assemblage structure

Most of the lithic clasts retrieved from excavated contexts are artifacts (~90% in the MW2-L3 and 100% in the small excavation of MW2-L4; Table 1). Debitage dominates the artifact categories in both assemblages. In MW2-L3, the only assemblage where the frequency of shaped tools (LCTs and retouched tools) can be quantified reliably, they constitute a minor fraction of the assemblage. The representation of shaped tools increases markedly in the younger MW2-L1&L2 assemblage.

### 3.2. Cores and the debitage

**Raw material.** The predominant lithology exploited in all the assemblages is of volcanic rocks, among which three texture grades of ignimbrite (‘regular’, glassy, and pumiceous) dominates. Constituting ≥50% of the raw materials represented, regular ignimbrite (hereafter ignimbrite) is dominant among cores, modified items, natural items, and indeterminate items recovered from MW2-L3, followed by varying proportions of other raw material types (Table 5; S1 Table). The combined frequency of the three ignimbrite types in MW2-L3 core assemblage is >90%, while it is ≥80% in MW2-L1&L2 (Table 5). This is consistent with the geological background, as flows of ignimbrite of various grades are extensively exposed along the banks of the Kawa, Asasa and Wabe Rivers, at distances from tens of meters up to 4–5 km from archaeological localities within the site-complex (S5 Fig; [1]). (See S1 Text for the differences between the petrographic characteristics of the three ignimbrite grades).

Unlike the cores, the raw material composition of debitage in the MW2-L3 assemblage is dominated by glassy ignimbrite (61% in the total debitage and >50% for specific debitage subcategories except large flakes; Table 2). Large flakes are made mostly of ignimbrite, whereas glassy ignimbrite is least represented (Table 2). A similar pattern is observed in the debitage assemblage of MW2-L4, where glassy ignimbrite dominates the various artifact components, but the small sample size prohibited quantitative treatment of the data.

**Table 2:** Absolute and relative frequencies of debitage breakdown (per raw materials) of MW2 assemblages.

**Debitage category**	**MW2-L4**																			
	Glassy ignimbrite				Ignimbrite		Pumiceous ignimbrite		Basalt			Scoria			Other volcanic			Total		
	*n*		%		*n*	%	*n*	%	*n*	%		*n*	%		*n*		%	*n*		%
Large flakes (>10 cm)	1		33.3		1	33.3	1	33.3	-	-		-	-		-		-	3		3.0
Small to medium-sized flakes (2–10 cm)	12		70.6		4	23.5	1	5.9	-	-		-	-		-		-	17		16.8
Micro-flakes (≤2 cm)	8		100.0		-	-	-	-	-	-		-	-		-		-	8		7.9
Broken flakes	5		71.4		2	28.6	-	-	-	-		-	-		-		-	7		6.9
Flake fragments (>2cm)	19		73.1		5	19.2	1	3.9	-	-		-	-		1		3.9	26		25.7
Debris (≤2 cm)	25		62.5		15	37.5	-	-	-	-		-			-		-	40		39.6
Total	70		69.3		27	26.7	3	3.0	-	-		-	-		1		1.0	101		
		**MW2-L3**																		
Large flakes (>10 cm)	4		8.33		34	70.83	10	20.83	-	-		-	-		-		-	48		0.84
Small to medium-sized flakes (2–10 cm)	449		51.97		371	42.94	21	2.43	22	2.54		1	0.11		-		-	864		15.17
Micro-flakes (≤2 cm)	284		76.75		67	18.11	-	-	17	4.59		-	-		2		0.54	370		6.49
Broken flakes	663		60.99		402	36.98	1	0.09	18	1.65		-	-		3		0.28	1087		19.08
Flake fragments (>2cm)	894		56.94		604	38.47	5	0.32	61	3.95		1	0.06		5		0.32	1570		27.56
Debris (≤2 cm)	1123		68.06		413	25.03	-	-	68	4.12		1	0.06		45		2.73	1650		28.97
Core fragments	54		50.47		45	42.05	2	1.87	6	5.61		-	-		-		-	107		1.88
Total	3471		60.94		1936	33.99	39	0.68	192	3.37		3	0.05		55		0.96	5696		
		**MW2-L1&L2**																		
Large flakes (>10 cm)	11			55.0	9	45.0	-	-	-		-	-		-	-	-		20	17.54	
Regular flakes (2–10 cm)	46			75.41	14	22.95	-	-	1		1.64	-		-	-	-		61	53.51	
Flake fragments (>2cm)	12			80.0	3	20.0	-	-	-		-	-		-	-	-		15	13.16	
Core fragments	8			44.4	8	44.4	2	11.1	-		-	-		-	-	-		18	15.79	
Total	77			67.54	34	29.82	2	1.75	1		0.88	-		-	-	-		114		

Glassy ignimbrite dominates the cores of MW2-L1&L2 (55%, *n* = 22; Table 5). Though not systematically excavated, trends seen within the debitage also closely track with the core exploitation pattern (Table 2). In contrast to patterns observed in the MW2-L3 debitage, large flakes in MW2-L1&L2 were also made mostly on glassy ignimbrite.

#### Core blanks

The types of blanks used as cores are informative of both technological decision-making during the knapping process as well as of economic behaviors involved in its implementation. MW2-L3 cores were dominantly made on cobbles/pebbles (67.6%, *n* = 175; Table 3; hereafter ‘cobble cores’), followed by flakes and angular elements (~17% and 12%, respectively; Table 3). The majority of the latter (*n* = 20) are relatively small-sized, whereas some items (*n* = 11) were made on large angular blocks (>1 kg).

**Table 3:** Absolute and relative frequencies (in parentheses) of core blank typologies (per raw materials) exploited by occupants of MW2.

Blank typology	MW2-L3						MW2-L1&L2			
	Glassy ign.	Ignimbrite	Pumice. ign.	Basalt	Scoria	Total	Glassy ign.	Ignimbrite	Basalt	Total
**Cobbles/pebbles**	36 (20.6)	96 (54.8)	25 (14.3)	14 (8.0)	4 (2.3)	175 (67.6)	13 (54.2)	7 (29.2)	4 (16.6)	24 (60.0)
**Angular elements**	19 (61.3)	10 (32.3)	2 (6.4)	-	-	31 (11.9)	7 (77.8)	2 (22.2)	-	9 (22.5)
**Flakes**	23 (51.1)	21 (46.7)	1 (2.2)	-	-	45 (17.4)	2(40.0)	3 (60.0)	-	5 (12.5)
**Slabs**	4 (50.0)	2 (25.0)	2 (25.0)	-	-	8 (3.1)	-	2 (100)	-	2 (5.0)
	82	129	30	14	4	259	22	14	4	40

Ign = ignimbrite.

More than half (~55%, *n* = 96; Table 3) of the cobble cores were made on ignimbrites, whereas cores made on angular elements were mostly made of glassy ignimbrite (~61%, *n* = 19; Table 3). As glassy ignimbrite is absent from the immediate vicinity of the MW2 locality, some of the latter cores (*n* = 6) may have been made on large angular fragments selected and transported to the MW2 area (see section 4.1). Three items in this group demonstrate also edge damage suggesting their additional use in heavy-duty percussion activities; the order of activities (i.e., their use as cores or as percussors) could not be established.

MW2-L1&L2 cores were mainly made on cobbles, angular elements being second in frequency (Table 3). Among cores made on angular elements, three were made on large angular blocks (>1 kg), of which two appear to have served as both cores as well as heavy-duty percussors. Similar to MW2-L3, the cores on large angular blocks were predominantly made on glassy ignimbrite. Cobble cores in the MW2-L1&L2 sample were also made on glassy ignimbrite, unlike the older assemblage.

#### Core size and reduction strategies

Raw material size affects the length of core use-life, namely, the length of the reduction sequence as well as the steps executed to achieve the knapper’s goals [132–134]. Based on our threshold criterion, cores in the two MW2 assemblages are associated with two separate reduction sequences—for small to medium-sized flake and for large flake blank production. Most of the cores in both MW2 assemblages are small to medium-sized flake cores, with only few cores distinguished as large flake cores (see section 3.4. below).

In MW2-L3, cores made on pumiceous ignimbrite are on average larger than those made on other raw material types, whereas cores made on glassy ignimbrite and basalt are smaller than others. These cores differ in mean length, width, and thickness according to their raw materials (Table 4) and the size differences are statistically significant based on the K-W test (length: H = 22.9, df = 3, *p* < 0.0001; width: H = 30.22, df = 3, *p* < 0.0001; thickness: H = 29.27, df = 3, *p* < 0.0001; scoria excluded due to small sample size). This is also true when only ignimbrite and glassy ignimbrite (the two dominantly exploited raw materials) are compared (M-W test: length: U = 3745.5, *p* < 0.0003; width: U = 3440.5, *p* < 0.0001; thickness: U = 3324, *p* < 0.0001).

**Table 4:** Descriptive statistics (means in bold) of dimensions of cores and natural items (per raw materials) in MW2 assemblages.

**Stat.**	**MW2-L3 Cores**														
	**Glassy ign.**			**Ignimbrite**			**Pumiceous ign.**			**Basalt**			**Scoria**		
	**L**	**W**	**Th**	**L**	**W**	**Th**	**L**	**W**	**Th**	**L**	**W**	**Th**	**L**	**W**	**Th**
*n*	81	81	81	129	129	129	30	30	30	14	14	14	4	4	4
**Mean**	**87.4**	**68.1**	**48.8**	**103.0**	**83.3**	**61.4**	**111.4**	**92.3**	**67.0**	**82.5**	**70.1**	**52.6**	**106.1**	**82.8**	**71.3**
**SD**	40.4	32.5	25.1	34.7	29.5	22.7	28.2	22.0	17.4	25.1	22.8	16.8	10.2	8.8	12.7
**Min**	27.4	25.3	12.8	40.1	34.3	25.7	58.4	49.7	32.6	51.3	39.5	25.8	96.1	76.8	58.9
**Max**	241.5	191.6	151.7	236.2	202.8	192.7	200.9	145.2	92.9	134.1	112.8	76.5	115.5	95.9	83.4
	**MW2-L1&L2 Cores**														
*n*	22	22	22	14	14	14	**-**	**-**	**-**	4	4	4	**-**	**-**	**-**
**Mean**	**93.6**	**84.4**	**58.8**	**135.4**	**111.5**	**72.0**	**-**	**-**	**-**	**60.9**	**77.7**	**57.9**	**-**	**-**	**-**
**SD**	38.5	34.8	30.1	43.8	18.4	21.2	**-**	**-**	**-**	20.3	15.4	19.5	**-**	**-**	**-**
**Min**	33.8	27.7	18.6	83.2	79.9	45.8	**-**	**-**	**-**	44.2	67.7	37.9	**-**	**-**	**-**
**Max**	164.9	141.0	132.1	224.5	141.1	105.4	**-**	**-**	**-**	85.7	100.3	83.4	**-**	**-**	**-**
	**MW2-L3 Natural items**														
*n*	4	4	4	59	59	59	29	29	29	7	7	7	10	10	10
**Mean**	**83.8**	**53.7**	**45.7**	**89.5**	**65.4**	**48.1**	**84.1**	**66.0**	**44.4**	**102.9**	**76.1**	**60.6**	**101.3**	**85.1**	**70.8**
**SD**	26.2	17.9	11.5	26.1	20.0	13.6	29.9	21.4	17.2	18.2	26.1	14.6	21.5	21.9	19.6
**Min**	57.7	38.1	31.2	46.1	35.6	25.9	42.0	37.3	21.3	86.2	41.8	43.8	56.2	40.8	27.0
**Max**	113.9	72.9	55.0	173.8	128.5	95.7	154.8	119.5	87.8	140.8	124.2	88.3	134.5	125.8	102.5

**Abbreviations:** Ign. = Ignimbrite; L = Length; W = Width; Th = Thickness; SD = Standard deviation

In MW2-L1&L2, size differences between cores made on glassy ignimbrite and ignimbrite (the two raw materials with sufficient sample size) are statistically different in mean length and mean width, but not in mean thickness (M-W test: length: U = 77, *p* = 0.01; width: U = 81, *p* = 0.01; thickness: U = 108, *p* = 0.13).

For the three types of ignimbrite in MW2-L3, the mean core dimensions are greater than those of natural items of the same raw material (Table 4). These results are statistically significant in the case of ignimbrite and pumiceous ignimbrite (ignimbrite: M-W test: length: U = 2868, *p = 0*.*006*; width: U = 2264, *p < 0*.*0001*; thickness: U = 2248.5, *p < 0*.*0001*; pumiceous ignimbrite: length: U = 192, *p = 0*.*0002*; width: U = 166, *p < 0*.*0001*; thickness: U = 154.5, *p < 0*.*0001*; natural items of glassy ignimbrite are too few for statistical comparison). By contrast, while the mean dimensions of basalt natural items in MW2-L3 are larger than those of basalt cores, the difference is statistically insignificant (length: U = 24, *p = 0*.*06*; width: U = 41, *p = 0*.*57*; thickness: U = 35, *p = 0*.*31*).

### 3.3. Reduction methods and intensity of small to medium-sized flake cores

Reduction methods of cores for small to medium-sized flakes in the MW2-L3 assemblage were used similarly for the three ignimbrite types. The bifacial abrupt partial (BAP) flaking method being utilized most frequently (Table 5; Fig 6D). For ignimbrite and glassy ignimbrite (constituting >80% of core raw materials), the BAP method is followed by the Multifacial and bifacial peripheral (BP) methods. Basalt cores were exploited relatively marginally (i.e., removals did not penetrate into the center of the knapped surface), most frequently by BP and Multifacial reduction methods. In the smaller core assemblage of MW2-L1&L2, cores were exploited mainly by the Multifacial method, followed by BP and BAP methods (Table 5).

**Table 5:** Absolute and relative frequencies (in parentheses) of core reduction strategies (per raw materials) employed by occupants of MW2.

Face interchange	Rotation	Surface exploitation	CRS	MW2-L3						MW2-L1&L2			
				Glassy ign.	Ignimbrite	Pumice. ign.	Basalt	Scoria	Total	Glassy ign.	Ignimbrite	Basalt	Total
Unifacial	Restricted	Peripheral	**USP**	-	-	-	-	-	-	-	-	-	-
Bifacial	Restricted	Peripheral	**BSP**	1 (1.2)	-	-	-	-	1 (0.4)	-	-	-	-
Unifacial	Restricted	Peripheral	**UAU1**	2 (2.4)	6 (4.6)	3 (10.0)	-	-	11 (4.2)	-	-	-	-
Unifacial	Restricted	Peripheral	**UAU2**	2 (2.4)	6 (4.6)	-	-	-	8 (3.1)	-	1 (7.1)	-	1 (2.5)
Unifacial	Full	Peripheral	**UAUT**	2 (2.4)	10 (7.8)	1 (3.3)	-	-	13 (5.0)	-	1 (7.1)	-	1 (2.5)
Unifacial	Full	Peripheral	**UABI**	3 (3.6)	8 (6.2)	3 (10.0)	1 (7.1)	-	15 (5.7)	-	1 (7.1)	-	1 (2.5)
Bifacial	Restricted	Peripheral	**BAP**	26 (31.7)	39 (30.2)	11 (36.6)	1 (7.1)	1 (25.0)	78 (30.1)	1 (4.5)	4 (28.6)	-	5 (12.5)
Bifacial	Restricted	Peripheral	**BALP**	1 (1.2)	2 (1.5)	1 (3.3)	-	-	4 (1.5)	-	1 (7.1)	-	1 (2.5)
Bifacial	Full	Peripheral	**BALT**	-	1 (0.8)	-	-	-	1 (0.4)	2 (9.1)	2 (14.2)	-	4 (10.0)
Unifacial	Full	Peripheral	**UP**	2 (2.4)	4 (3.1)	1 (3.3)	-	-	7 (2.7)	2 (9.1)	-	-	2 (5.0)
Bifacial	Full	Peripheral	**BP**	12 (14.6)	19 (14.7)	7 (23.3)	6 (42.9)	1 (25.0)	45 (17.4)	4 (18.1)	3 (21.4)	-	7 (17.5)
Unifacial	Full	Peripheral	**UC**	3 (3.6)	2 (1.5)	1 (3.3)	-	-	6 (2.3)	-	-	-	-
Bifacial	Full	Central	**BHC**	2 (2.4)	-	-	-	-	2 (0.7)	2 (9.1)	-	1 (25.0)	3 (7.5)
Bifacial	Full	Central	**DISCOID**	5 (6.1)	2 (1.5)	1 (3.3)	-	-	8 (3.1)	2 (9.1)	-	-	2 (5.0)
		Unstructured	**POLYHEDRAL**	3 (3.6)	3 (2.3)	-	-	-	6 (2.3)	-	-	-	-
		Unstructured	**MULTIFACIAL**	18 (21.9)	27 (20.9)	1 (3.3)	6 (42.9)	2 (50.0)	54 (21.8)	9 (40.9)	1 (7.1)	3 (75.0)	13 (32.5)
				**82 (31.7)**	**129 (49.8)**	**30 (11.6)**	**14 (5.4)**	**4 (1.5)**	**259**	**22 (55.0)**	**14 (35.0)**	**4 (10.0)**	**40**

**Abbreviations: CRS** = Core Reduction Scheme; **USP** = Unifacial simple (<45°) partial exploitation; **BSP** = Bifacial simple partial exploitation; **UAU1** = Unidirectional abrupt (>45°) unifacial exploitation on one knapping surface; **UAU2** = Unidirectional abrupt unifacial exploitation on two independent knapping surfaces; **UAUT** = Unifacial abrupt unidirectional total exploitation; **UABI** = Unifacial abrupt bidirectional exploitation; **BAP** = Bifacial abrupt partial exploitation; **BALP** = Bifacial alternating partial exploitation; **BALT** = Bifacial alternating total exploitation; **UP** = Unifacial peripheral exploitation; **BP** = Bifacial peripheral; **UC** = Unifacial centripetal exploitation; **BHC** = Bifacial hierarchical centripetal; **Discoid** = this method is similar to BHC, except for the unclear hierarchization of surfaces; **Polyhedral** = cores with three or more knapping surfaces, which became spherical as reduction sequence continued and covered the whole surface of the core; **Multifacial** = cores with three or more knapping surfaces with no clear organization of flaking.

Overall, the MW2 core assemblages contain higher percentages of cores manifesting bifacial exploitation than of cores with single surface exploitation. Counted together, cores with BAP, BP, bifacial alternating partial (BALP), bifacial alternating total (BALT), bifacial hierarchical centripetal (BHC), and Discoid reduction systems constitute 52.7% (*n* = 140) in MW2-L3 and 55% (*n* = 22) in MW2-L1&L2 assemblages (Table 5; Fig 6D, 6F–6H). This suggests bifacial knapping techniques were favorably applied in both MW2 assemblages. In the ~1.6 Ma-old assemblage of MW2-L3, bifacially reduced cores exhibit mainly peripheral exploitation (e.g., BAP and BALP methods constitute about 60% of the bifacially reduced cores; Table 5), suggesting shorter core use lives. In the younger assemblages of MW2-L1&L2, core use lives were extended by rotating the exploitation surfaces as well as by exploiting the central volumes of cores (e.g., BALT, BP, BHC, and Discoid methods account for about 73% of the bifacially reduced cores; Table 5).

Additionally, reduction methods restricted to a core’s single surface account for a significant proportion of cores of MW2-L3 (23.7%, *n* = 63; Table 5; Figs 6E and 7E). Their proportion decreases to only 12.5% (*n* = 5) in the younger MW2-L1&L2 core assemblage. Of the total cores in this category, 66.7% (*n* = 42) in MW2-L3 and 83.3% (*n* = 5) in MW2-L1&L2 assemblages exhibit of a single knapping surface. In both assemblages, most of the cores in this category underwent long exploitation sequences along the reduction plane, either by fully rotating the exploitation surface (e.g., unifacial abrupt unidirectional total [UAUT], unifacial peripheral [UP], and unifacial centripetal [UC] methods) or by bidirectional knapping of the surface (e.g., unifacial abrupt bidirectional [UABI] method).

**Fig 5:**
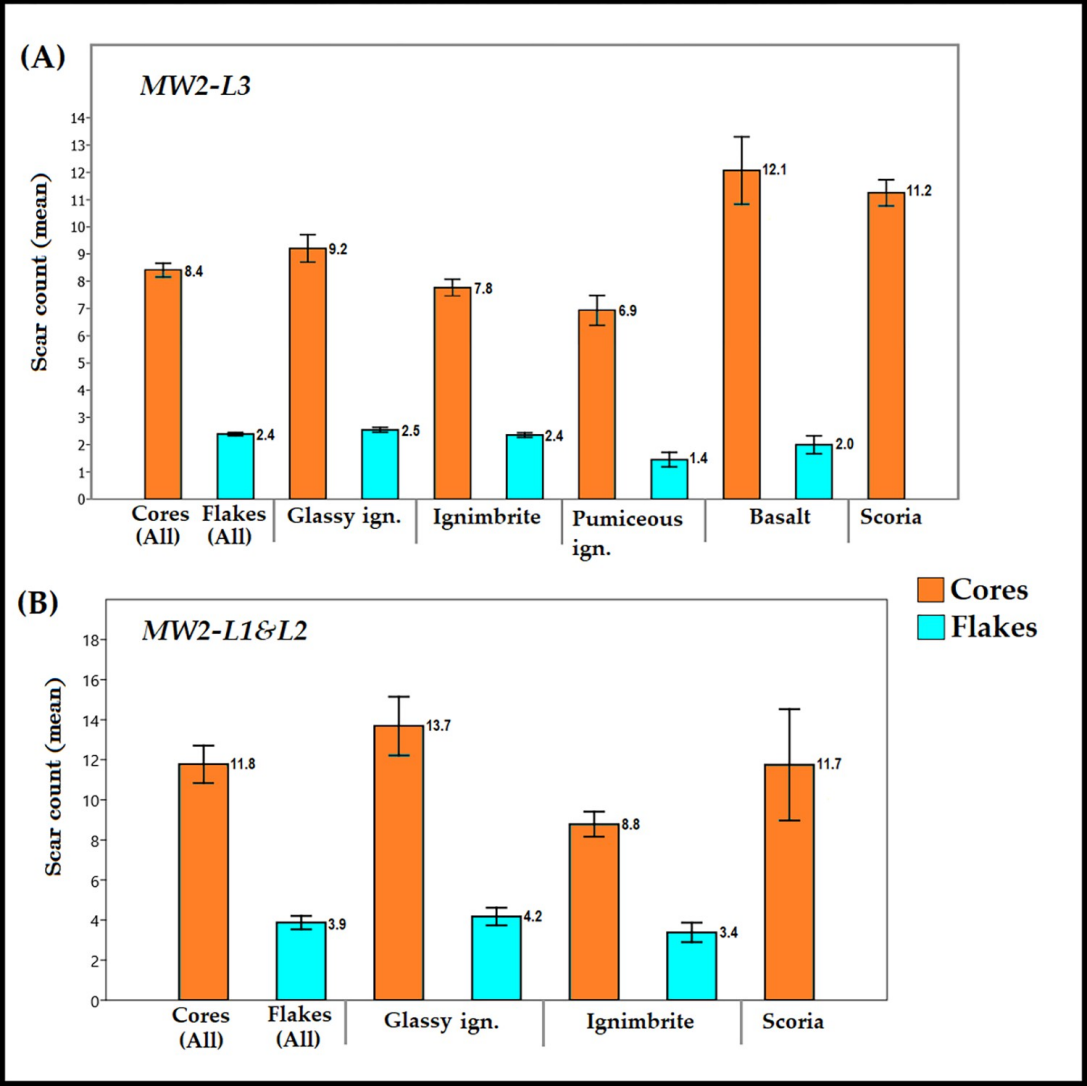
Box plot (with standard error) showing the average scar counts on the surface of cores and flakes (as per raw materials represented) from A) MW2-L3 and B) MW2-L1&L2.

Intra-assemblage comparison of core exploitation patterns reveals some differences in the treatment of raw materials in each assemblage. In MW2-L3, the marginally represented basalt and scoria appear to be more intensively exploited than the variants of ignimbrite (Fig 5A). This trend is reversed in the MW2-L1&L2 core assemblage, where cores made on the dominant raw material (glassy ignimbrite) are the most intensively exploited (Fig 5B). In MW2-L3, glassy ignimbrite is more intensively exploited than the other two ignimbrite types (K-W test: total scar count: H = 7.637, df = 2, *p = 0*.*02*). This differential treatment of the raw materials is manifest also by core-to-flake ratio in the *in situ* MW2-L3 assemblage, which is much higher for glassy ignimbrite (1:4.0) than for ignimbrite (1:2.6), pumiceous ignimbrite (1:0.7) or basalt (1:1.57), as well as the average for the whole core sample (1:2.7). (Flakes associated with LCTs *façonnage* phases were excluded from the analysis as they belong to the LCTs reduction sequence; these items are described below).

**Fig 6:**
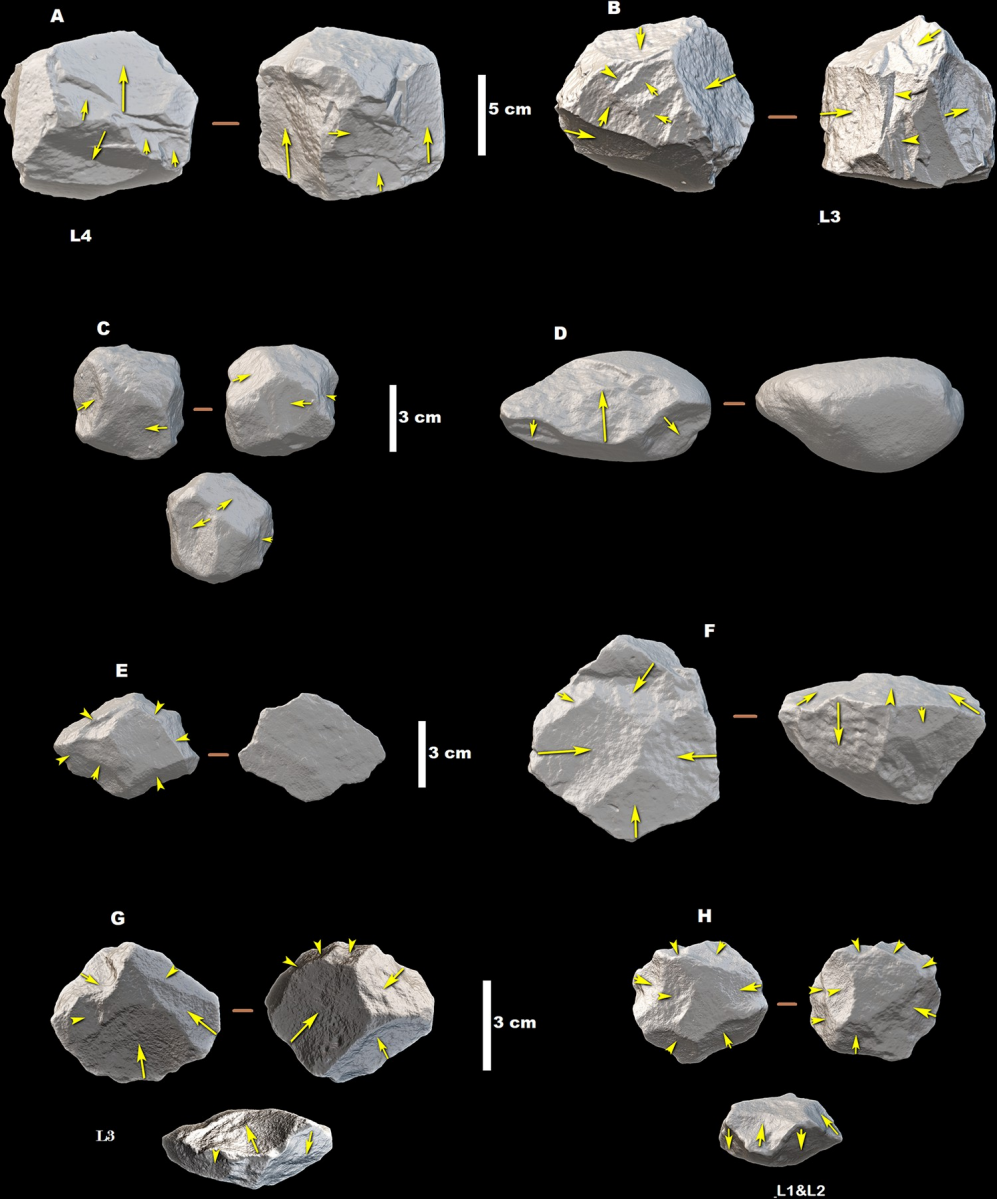
Cores from the small to medium-sized flaking system from MW2-L4 (A), MW2-L3 (B–G), and MW2-L1&L2 (H); (A), (B), and (C) are Multifacial cores; (D) Bifacial abrupt partial (BAP) exploited core; (E) Core on flake; (F) Bifacial hierarchical centripetal (BHC) exploited core; (G) and (H) are Discoids. (A), (B), (E), (G), and (H) are made on glassy ignimbrite, (C) and (F) are made on ignimbrite, and (D) is made on basalt. The arrows indicate direction of flaking.

The intensity of core exploitation, as deduced from the scar numbers on core surfaces (Fig 5), differs between the older and younger MW2 assemblages discussed here. The overall mean number of scars on MW2-L1&L2 cores (11.77 scars) constitutes a 40% increase compared to MW2-L3 cores (8.41 scars). This diachronic increase is higher for cores made on glassy ignimbrite (48.5%) compared to only about 13% for cores made on ignimbrite.

De la Torre [8] considers that BHC, Discoid, Polyhedral, and Multifacial cores manifest ‘efficient’ maintenance of knapping surfaces, which can be explained by sustained exploitation of the pre-existing flaking angles on a given core. By this definition, MW2 cores demonstrate a diachronic increase in the ‘efficiency’ of exploitation, from about 28% (*n* = 70) of these methods in the whole core sample of MW2-L3, to about 45% (*n* = 17) in MW2-L1&L2 (Table 5). The ‘efficiently’ exploited cores also exhibit parallel diachronic changes in terms of organization of the flaking sequence, i.e., structured *vs*. unstructured exploitation. The proportion of ‘efficient’ cores with unstructured surface exploitation patterns (i.e., Multifacial and Polyhedral cores; Fig 6B and 6C) increased from ~24% in MW2-L3 to ~33% in MW2-L1&L2, while that of cores with structured exploitation of central surface (i.e., BHC and Discoid cores; Fig 6F–6H) increased from ~4% in MW2-L3 to ~13% in MW2-L1&L2 (Table 5).

We use the term ‘regular’ flakes to distinguish flakes whose characteristics cannot be linked to specific technological procedures of LCT production. The majority of small to medium-sized flakes in MW2-L3 are ‘regular’ flakes (81.6%, *n* = 705) made of ignimbrite and glassy ignimbrite (>90% of the entire assemblage; Table 6). Unlike the pattern observed in the cores (Table 5), the two raw materials occur in comparable proportions among the regular flakes (ca. 47% each; Table 6). However, glassy ignimbrite predominates among the small to medium-sized flakes in MW2-L3 that are associated with LCTs shaping (≥ 75% in each category; Table 6). This is the case also for both the regular and LCTs related flakes in the relatively small assemblage of MW2-L1&L2 (Table 6).

**Table 6:** Breakdown of numbers of small to medium-sized flakes in MW2 assemblages broken down to raw material categories.

Flake typology	**MW2-L3**											
	**Glassy ign.**		**Ignimbrite**		**Pumiceous ign.**		**Basalt**		**Scoria**		**% in total assemblage**	
	*n*	%	*n*	%	*n*	%	*n*	%	*n*	%		
**Regular flakes**	329	46.7	332	47.1	21	2.9	22	3.1	1	0.1	81.6% (*n* = 705)	
**LCTs shaping flakes**												
Roughing-out flakes	6	75.0	2	25.0	-	-	-	-	-	-	8	5.0%
Thinning flakes	75	70.8	31	29.2	-	-	-	-	-	-	106	66.7%
Finishing flakes	39	86.7	6	13.3	-	-	-	-	-	-	45	28.3%
% in the category	120	75.5	39	24.5	-	-	-	-	-	-	18.4% (*n* = 159)	
Total assemblage	**449**		**371**		**21**		**22**		**1**		**864**	
	**MW2-L1&L2**											
**Regular flakes**	23	63.9	12	33.3	-	-	1	2.8	-	-	59.1% (*n* = 36)	
**LCTs shaping flakes**												
Roughing-out flakes	18	90	2	10	-	-	-	-	-	-	20	80.0%
Thinning flakes	3	100	-	-	-	-	-	-	-	-	3	12.0%
Finishing flakes	2	100	-	-	-	-	-	-	-	-	2	8.0%
% in the category	23	92.0	2	8.0	-	-	-	-	-	-	40.9% (*n* = 25)	
Total assemblage	**46**		**14**		**-**		**1**				**61**	

Ign. = Ignimbrite

The majority of regular as well as large flakes in MW2-L3 are end-struck (65.9% and 58.3%, respectively; Table 7). Likewise, LCTs shaping flakes in MW2-L3 are also predominantly end-struck (Table 7). Yet, LCT finishing flakes differ in that nearly 50% of them are side-struck. The pattern is similar for both regular and large flakes, as well as LCT roughing out flakes from MW2-L1&L2 (Table 7).

**Table 7:** Absolute and relative frequencies (in parentheses) of the technological features of flake categories in the MW2 assemblages.

Technological attributes	MW2-L3					MW2-L1&L2				
	Regular flakes	Large flakes	LCT shaping flakes			Regular flakes	Large flakes	LCT shaping flakes		
			*Roughing*	*Thinning*	*Finishing*			*Roughing*	*Thinning*	*Finishing*
***Flaking direction***										
End-struck	**464** (65.9)	**28** (58.3)	**6** (75.0)	**77** (72.6)	**23** (51.1)	**20** (55.6)	**13** (65.0)	**12** (60.0)	**2** (66.7)	-
Side-struck	**222** (31.5)	**18** (37.6)	**1** (12.5)	**27** (25.5)	**21** (46.7)	**11** (30.6)	**6** (35.0)	**7** (35.0)	-	**2** (100)
Special side-struck	**17** (2.4)	**2** (2.1)	**1** (12.5)	**2** (1.9)	**1** (2.2)	**4** (11.1)	**1** (5.0)	**1** (5.0)	**1** (33.3)	-
Indeterminate	**1** (0.1)	-	-	-	-	**1** (2.8)	-	-	-	-
***Striking platform***										
Cortical	**17** (2.4)	**3** (6.2)	-	**1** (0.9)	**1** (2.2)	**2** (5.6)	-	**1** (5.0)	-	-
Punctiform	**66** (9.4)	**4** (8.3)	-	-	**3** (6.7)	**4** (11.1)	**1** (5.0)	-	-	**1** (50.0)
Plain	**534** (75.8)	**35** (72.9)	**8** (100)	**95** (89.6)	**32** (71.1)	**26** (72.2)	**13** (65.0)	**17** (85.0)	**2** (66.7)	-
Dihedral	**35** (4.9)	**1** (2.1)	-	**8** (7.6)	**8** (17.8)	-	**1** (5.0)	-	**1** (33.3)	**1** (50.0)
Removed	**28** (3.9)	**4** (8.3)	-	-	-	**3** (8.3)	**2** (10.0)	-	-	-
Crushed	**21** (2.9)	**1** (2.1)	-	**2** (1.9)	**1** (2.2)	-	**1** (5.0)	**1** (5.0)	-	-
Missing	**3** (0.4)	-	-	-	-	**1** (2.8)	**2** (10.0)	**1** (5.0)	-	-
***Dorsal scar pattern***										
Cortical	**23** (3.3)	**1** (2.1)	-	**1** (0.9)	**1** (2.2)	**1** (2.8)	-	**1** (5.0)	-	-
Unidirectional	**290** (41.2)	**7** (14.6)	**1** (12.5)	**53** (50.0)	**17** (37.8)	**11** (30.6)	**6** (35.0)	**4** (20.0)	**1** (33.3)	-
Opposed	**136** (19.3)	**18** (37.5)	**1** (12.5)	**20** (18.9)	**4** (8.9)	**10** (27.8)	**8** (40.0)	**3** (15.0)	**1** (33.3)	-
Orthogonal	**160** (22.7)	**17** (35.4)	**4** (50.0	**25** (23.5)	**17** (37.8)	**10** (27.8)	**5** (25.0)	**11** (55.0)	-	**2** (100)
Centripetal	**49** (6.9)	**5** (10.4)	**2** (25.0)	**4** (3.8)	**2** (4.4)	**4** (11.1)	**1** (5.0)	**1** (5.0)	**1** (33.3)	-
Indeterminate	**46** (6.5)	-	-	**3** (2.8)	**4** (8.9)	-	-	-	-	-
**Total**	**704**	**48**	**8**	**106**	**45**	**36**	**20**	**20**	**3**	**2**

Plain striking platforms predominate among regular flakes, large flakes and LCT shaping flakes in MW2-L3 and MW2-L1&L2 (Table 7). Other platform types are sporadically and marginally represented in both MW2-L3 and MW2-L1&L2 (Table 7). The scar patterns on the dorsal faces of regular flakes, as well as thinning and finishing flakes MW2-L3, are mainly unidirectional, orthogonal, and opposed, appearing in variable proportions among the various flake types (Table 7). The same scar patterns predominate also in the large flake component of the same assemblage, with the difference that orthogonal and opposed scar patterns are more frequent than unidirectional ones. In general, the distribution of scar patterns in MW2-L1&L2 is similar to that observed in MW2-L3 flake assemblage.

### 3.4. Technological characteristics of large cutting tools: Debitage and façonnage

**Fig 7:**
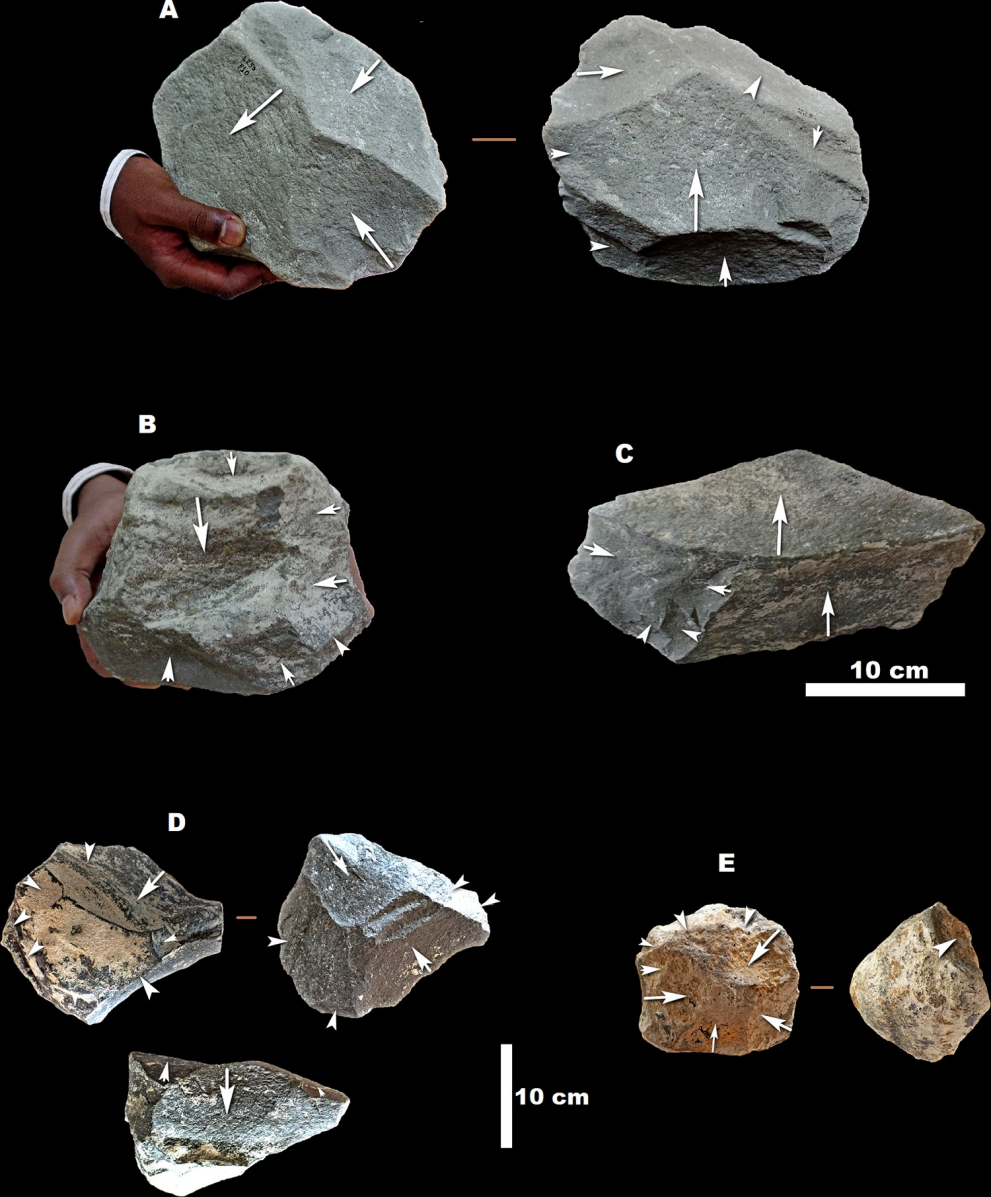
Large cores (A–D) and small debitage (E) cores from MW2-L3: A) Multifacial core (8 kg); B) Multifacial core (5.2 kg); C) Multifacial core (6 kg); D) Multifacial core (2.4 kg); E) Unifacial Centripetal (UC) exploited core. (A) and (C) are made on ignimbrite, (B) and (D) are made on glassy ignimbrite, and (E) is made on pumiceous ignimbrite. The arrows indicate direction of flaking.

The second reduction process in the MW2 assemblages was designed for the production of large flakes that could then be modified into LCTs. Based on the criteria set in this study, few cores can be defined as large cores (2.26%, *n* = 6, and 2.32%, *n* = 1, in MW2-L3 and MW2-L1&L2, respectively; Fig 7; S7C & S7D Fig). In MW2-L3, the average largest dimension of dominant flake scars on such cores is 107.8 mm and they were reduced by Multifacial (*n* = 3), UAU2 (*n* = 1), BAP (*n* = 1), and BP (*n* = 1) reduction methods. The maximum dimension of the dominant scar on a single core for large flakes in MW2-L1&L2 is 107.9 mm, flaked using Multifacial reduction method.

In MW2-L3, the relative frequency of ignimbrite among large cores is high compared to the total assemblage. Likewise, large flakes in this assemblage are predominantly (70.8%, n = 34; Table 2) made on ignimbrite. Size differences of the MW2-L3 large flakes made on the three types of ignimbrite are also minor (S2 Table). These differences are not significant for ignimbrite and pumiceous ignimbrite in the MW2-L3 assemblage (M-W test: length: U = 167, *p = 0*.*94*; width: U = 108, *p = 0*.*08*; thickness: U = 158, *p = 0*.*74*; the sample of glassy ignimbrite was too small for quantitative analysis).

The single large core from MW2-L1&L2 is made on glassy ignimbrite. In this assemblage, glassy ignimbrite dominates the large flakes (55%, *n* = 11), followed by ignimbrite (Table 2). The latter flakes are larger on average (specially in mean length and width), but the differences are not statistically significant (M-W test; length: U = 28, *p = 0*.*11*; width: U = 35, *p = 0*.*28*; thickness: U = 42, *p = 0*.*59*). Large flakes made on glassy ignimbrite are significantly smaller than the handaxes and picks in the assemblage that are made on the same raw material (K-W test; length: H = 15.53, *p = 0*.*0004*).

LCTs in the MW2-L3 were dominantly made on glassy ignimbrite (Table 8). This markedly contrasts with the raw material exploitation of both small debitage cores and large cores, as well as the large flakes in this assemblage where ignimbrite dominates. On the other hand, both LCTs and large flakes in the MW2-L1&L2 were dominantly made on glassy ignimbrite (Table 8; S2 Table).

**Table 8:** Physical and technological attributes of LCTs in the MW2 assemblages.

Attributes	Picks				Crude LCTs/handaxes				Large scrapers		Cleavers	
	MW2-L3		MW2-L1&L2		MW2-L3		MW2-L1&L2		MW2-L3		MW2-L1&L2	
	*n*	*%*	*n*	*%*	*n*	*%*	*n*	*%*	*n*	*%*	*n*	*%*
**Raw material**												
Glassy ignimbrite	2	100.0	6	100.0	3	75.0	26	89.7	5	83.3	1	100.0
Ignimbrite	-	-	-	-	1	25.0	3	10.3	1	16.7	-	-
**Blank typology**												
Flake	1	50.0	4	66.7	4	100	27	93.1	6	100.0	1	100.0
Cobble	-	-	-	-	-	-	1	3.4	-	-	-	-
Indeterminate	1	50.0	2	33.3	-	-	1	3.4	-	-	-	-
**Flaking direction**												
Side-struck	-	-	1	25.0	2	50.0	5	18.5	3	50.0	-	-
Special side-struck	-	-	2	50.0	1	25.0	5	18.5	1	16.7	1	100.0
End-struck	-	-	1	25.0	1	25.0	6	22.2	2	33.3	-	-
Indeterminate	1	100.0	-	-	-	-	11	40.7	-	-	-	-
**Striking platform**												
Plain	-	-	3	50.0	2	50.0	11	40.7	5	83.3	1	100.0
Cortical	-	-	-	-	-	-	1	3.7	-	-	-	-
Removed	1	50.0	1	16.7	2	50.0	11	40.7	1	16.7	-	-
Indeterminate	1	50.0	2	33.3	-	-	4	14.8	-	-	-	-
**Total**	**2**		**6**		**4**		**29**		**6**		**1**	

#### The production system of LCT blanks

The identification of production methods of flake blanks of LCTs in the MW2 assemblages is facilitated by the overall low investment in the shaping of LCTs, which allows recognition of flake striking direction and original scar patterns. At least three production methods of large flakes, detached from giant cores (see S1 Text), can be inferred.

The majority of LCTs in both MW2 assemblages were made on flakes. LCTs on cobbles make a rare appearance only in MW2-L1&L2 (Table 8). In the MW2-L3 assemblage, side-struck (including special side-struck) flakes were used preferentially as blanks of LCTs (Table 8). In MW2-L1&L2, the blanks of handaxes are mostly ‘indeterminate’ because the characteristics of the original blanks are obscured by shaping (see section below); where direction of blow could be observed, side-struck flakes outnumber end-struck ones.

Post-detachment shaping (façonnage)

In flake-based LCTs, several attributes serve to evaluate the amount of post-detachment shaping, including the types of striking platform. The common striking platform of LCTs in the MW2 assemblages is ‘plain’, followed by intentionally flaked-off (‘removed’) and ‘indeterminate’ (where the original flake platform is heavily modified in the ventro-proximal area of the flake) platforms (Table 8).

**Fig 8:**
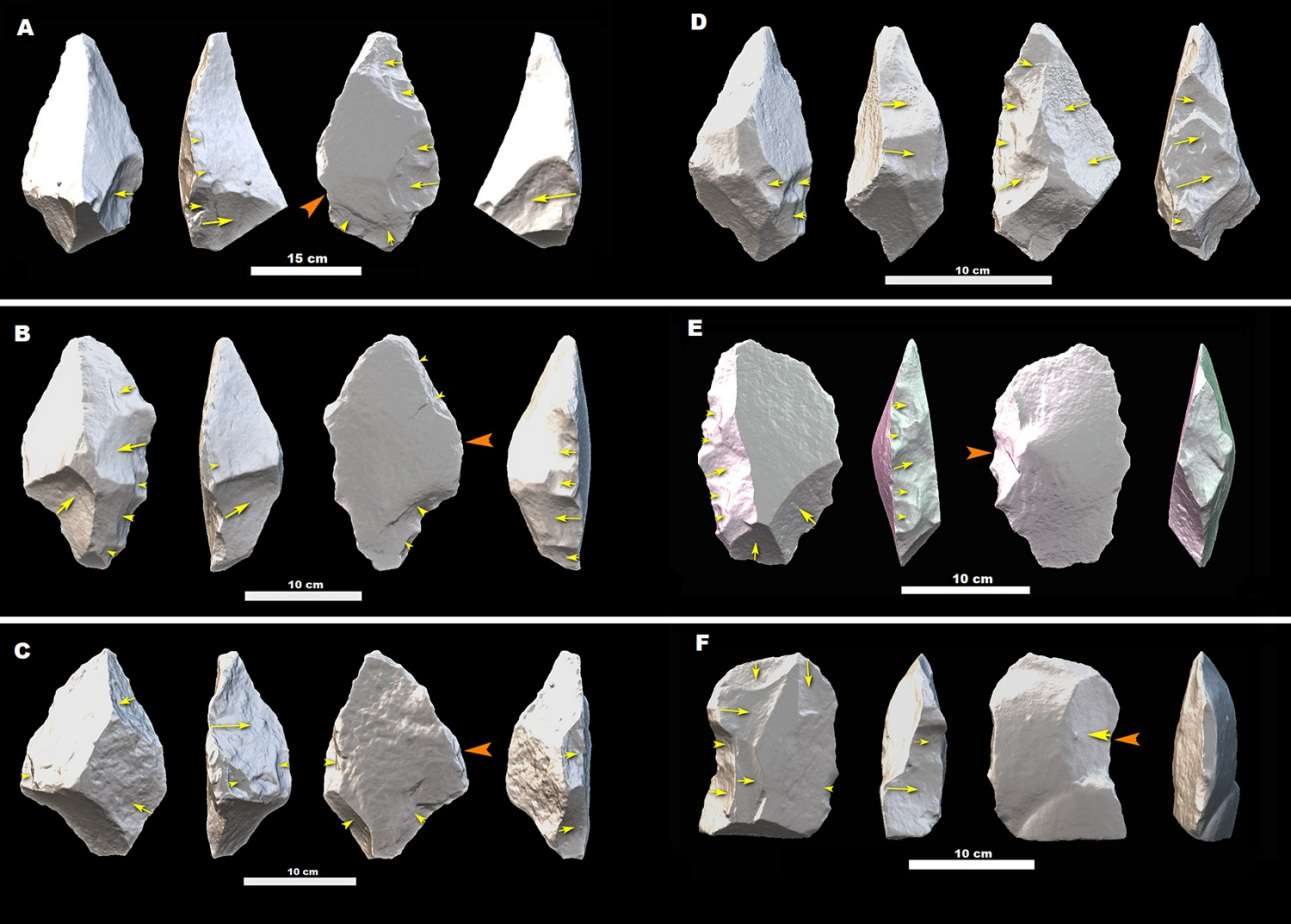
Crude LCTs (A–C), Pick (D), and Large scrapers/knives (E and F) from MW2-L3. (A) is made on special side-struck flake, (B), (C), (E) and (F) are on side-struck flake, and the blank of the pick (D) is indeterminate. All of the tools are made on glassy ignimbrite. Orange arrows indicate the direction of flaking of the blanks, while yellow arrows indicate the direction of removals during shaping phases.

**Fig 9:**
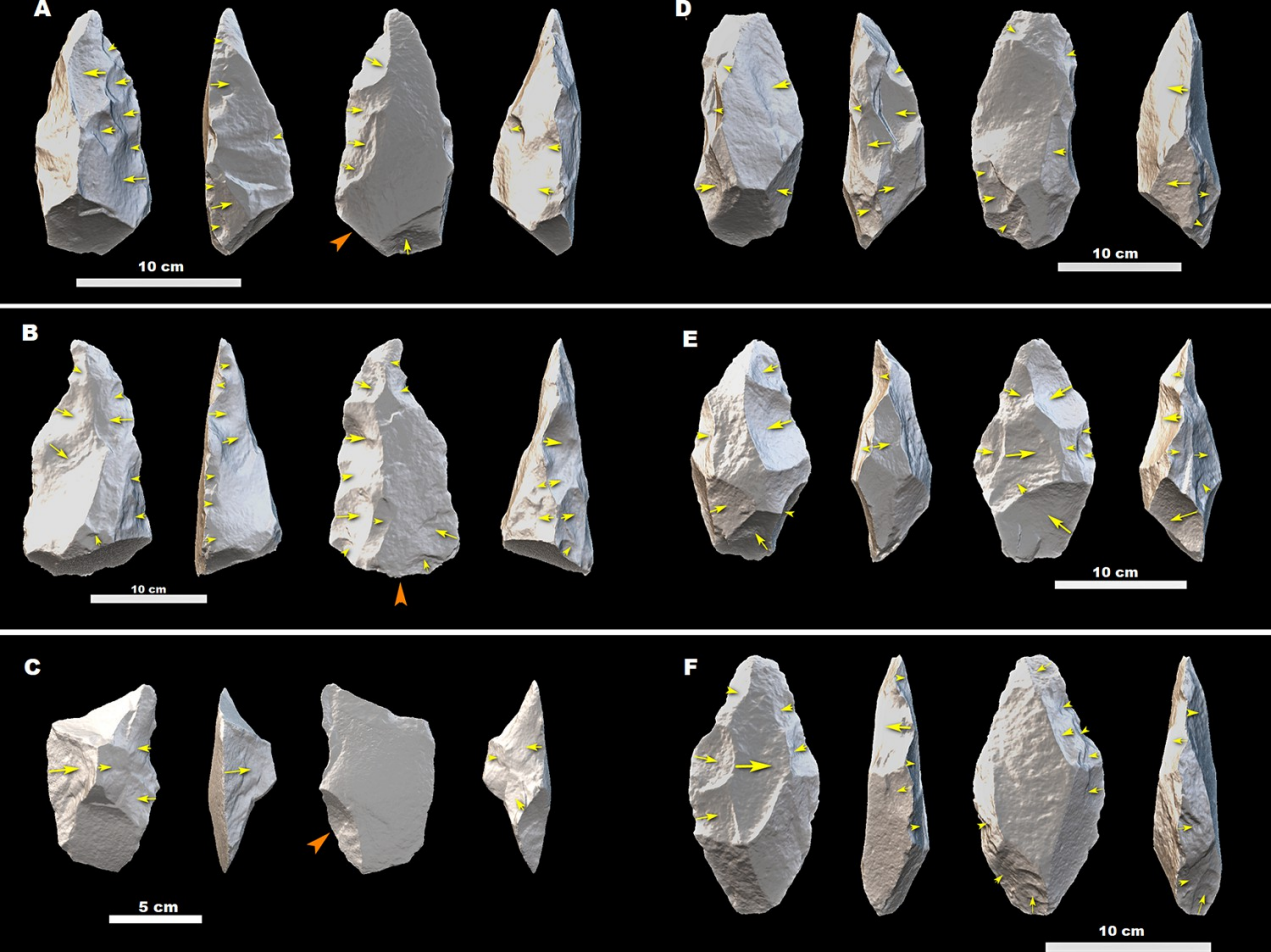
Trihedral picks (A and B), Cleaver (C), and Handaxes (D–F) from MW2-L1&L2. The picks and a cleaver are made on flakes (the arrows indicate the direction of flaking of flake blanks), whereas the blanks of the handaxes are indeterminate. All tools are made on glassy ignimbrite. Orange arrows indicate the direction of flaking of the blanks, while yellow arrows indicate the direction of removals during shaping phases.

**Fig 10:**
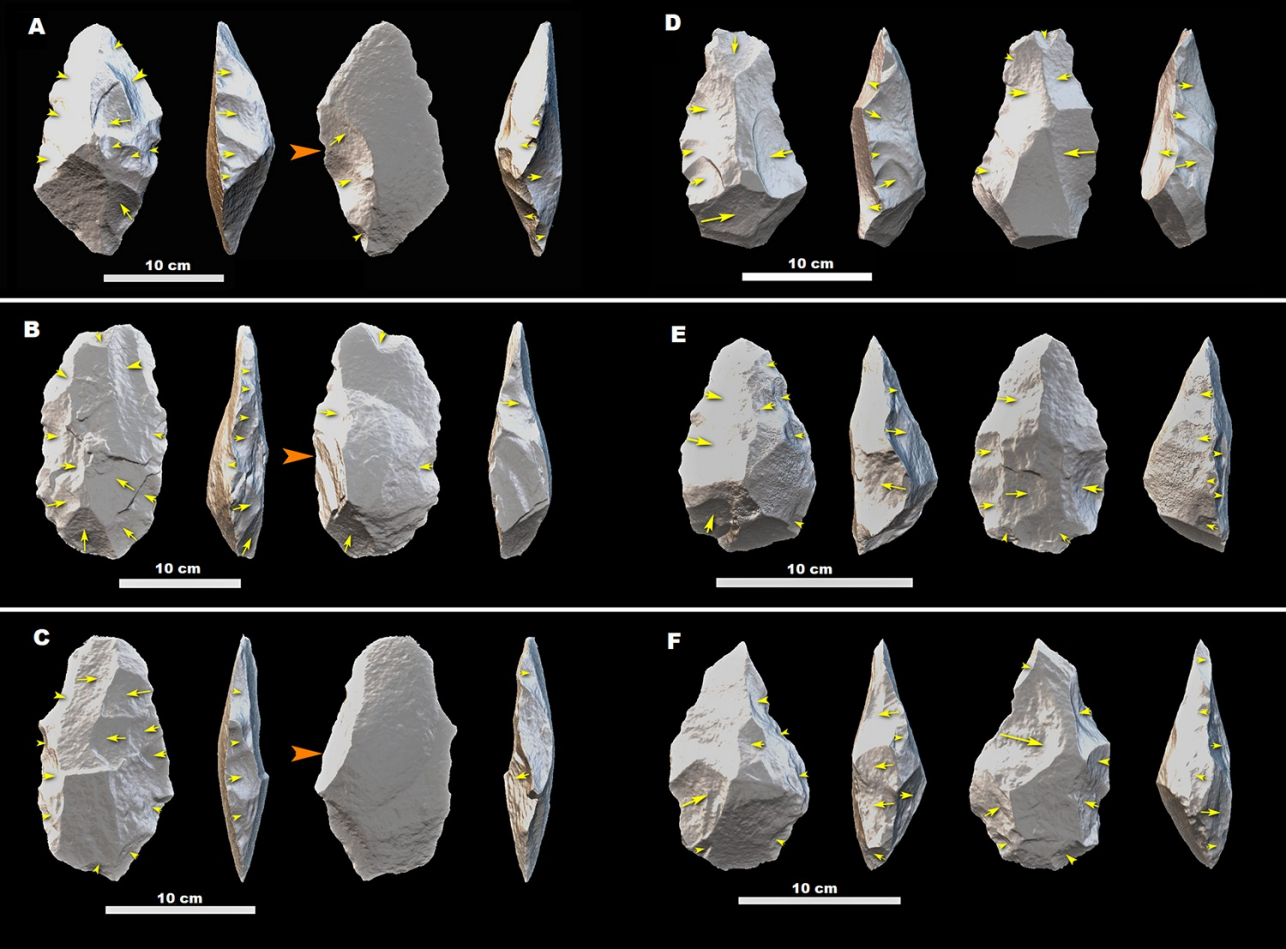
Handaxes from MW2-L1&L2. (A), (B), and (C) are made on side-struck flake blanks (see the direction of the arrows), while the blanks of (D), (E), and (F) are indeterminate. All tools are made on glassy ignimbrite. Orange arrows indicate the direction of flaking of the blanks, while yellow arrows indicate the direction of removals during shaping phases.

All the crude LCTs (*n* = 4) from MW2-L3 weigh >1 kg. With a mean weight of 1.93 kg, these items are heavier than bifacial items from MW2-L1&L2, as well as from picks and large scrapers in both assemblages (Table 9). Crude bifacial items are also the largest of the LCT types represented in the MW2-L3 assemblage (Table 9), preserving very thick profiles (Table 10; Fig 8A–8C). MW2-L1&L2 handaxes are both relatively thinner and more elongated than crude LCTs in MW2-L3 (Table 10; Figs 9 and 10). While the average thickness of picks in the two assemblages is similar (Table 9), MW2-L1&L2 picks are more elongated (Table 10).

**Table 9:** Descriptive statistics of dimensions of LCTs in MW2 assemblages.

Stat.	Crude LCTs/handaxes								Picks								Large scrapers			
	MW2-L3				MW2-L1&L2				MW2-L3				MW2-L1&L2				MW2-L3			
	L	W	Th	We.	L	W	Th	We.	L	W	Th	We.	L	W	Th	We.	L	W	Th	We.
*n*	4	4	4	4	29	29	29	29	2	2	2	2	6	6	6	6	6	6	6	6
**Mean**	217.2	138.9	99.4	1932.5	162.3	91.6	50.1	603.5	140.2	77.2	61.5	426.4	179.2	79.4	60.8	655.4	119.2	110.2	48.9	668.9
**S.D.**	52.5	20.8	38.6	1080.1	28.6	14.4	12.1	385.7	8.9	3.2	0.9	80.4	22.7	21.3	12.9	365.0	34.9	36.9	11.7	325
**Min**	180.9	112.2	70.8	1230	108.7	72.7	32.9	226.1	133.9	74.9	60.8	369.5	149.3	60.8	45.7	268	81.6	72.9	26.8	204.9
**Max**	294.5	158.3	154.9	3510	258.6	149.8	86.2	2365	146.5	79.5	62.2	483.2	206.7	113.7	81.8	1142	177.3	155.3	61.1	1048

**Abbreviations:** Ign. = Ignimbrite; L = Length; W = Width; Th = Thickness, We. = weight

**Table 10:** Descriptive statistics of the relative thickness, elongation, and total scar frequency of picks and handaxes from MW2 assemblages.

Statistics	Picks						Crude LCTs/handaxes					
	MW2-L3			MW2-L1&L2			MW2-L3			MW2-L1&L3		
	Th/W	W/L	Scar mean	Th/W	W/L	Scar mean	Th/W	W/L	Scar mean	Th/W	W/L	Scar mean
*n*	2	2	2	6	6	6	4	4	4	29	29	29
**Mean**	0.797	0.550	11	0.774	0.337	16	0.702	0.65	12.5	0.550	0.57	14.6
**S.D.**	0.02	0.01	4.24	0.06	0.04	2.19	0.18	0.14	4.3	0.11	0.06	6.1
**Min**	0.782	0.542	8	0.719	0.290	13	0.568	0.537	10	0.345	0.448	6
**Max**	0.811	0.559	14	0.865	0.395	19	0.978	0.840	19	0.758	0.688	25

**Abbreviations:** Th/W: thickness/width (Relative thickness index); W/L: width/length (Elongation index)

Sample size limitations of LCTs in the MW2 assemblages render further quantitative analysis unwarranted.

In the MW2 assemblages, the mean number of scars on the dorsal face is typically higher than on the ventral face of any given bifacial item. As the definition of ‘ventral’ in most instances overlaps with the original blank ventral face, this observation suggests a relatively higher investment in the dorsal face, possibly in an attempt to further reduce the thick profiles of the blanks (S3 Table).

The number of scars on each face increases from the older to the younger MW2 assemblages, but the change is not homogenous for all the types. The ventral faces of large scrapers from MW2-L3 are barely retouched/transformed during the shaping phase (S3 Table). Compared to MW2-L3 crude LCTs, the mean number of scars on MW2-L1&L2 handaxes increased by only 8.4% for the dorsal face and to 32.6% for the ventral face (S3 Table). This shows the greater attention given to shaping the ventral faces of handaxes over time. In contrast, investment in the shaping of picks continuously focused on the dorsal face, with mean number of scars increasing from MW2-L3 to MW2-L1&L2 by 79.9% and 4% on the dorsal and ventral faces, respectively.

Flakes associated with the shaping (*façonnage*) of LCTs form the second most frequent category of small to medium-sized flakes in both MW2-L3 and MW2-L1&L2 assemblages (Table 6; S1 Text; S3 Fig). Their proportion also showed marked increase over time, i.e., from ~18% in MW2-L3 to ~41% in MW2-L1&L2 (Table 6). However, the frequencies of technological flakes differ between the MW2 assemblages. Thinning flakes dominate (66.7%) in MW2-L3, while roughing-out flakes are least represented (5%; Table 6). In contrast, in the MW2-L1&L2 assemblage roughing-out flakes are the dominant flake type associated with façonnage (80%; Table 6). (Note that we are unable to confidently rule out size-related collection bias).

### 3.5. Hammerstones

**Fig 11:**
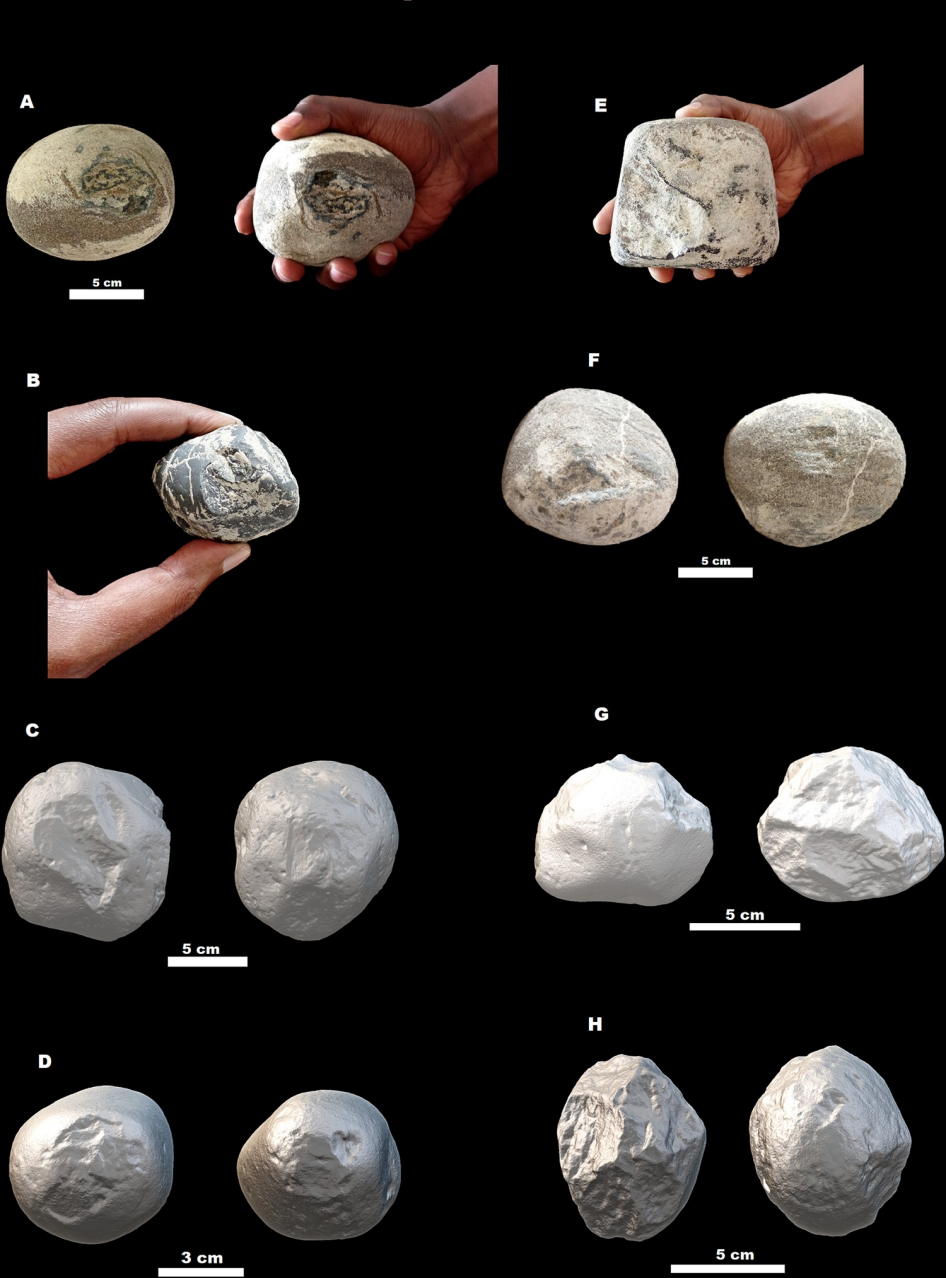
Hammerstones from MW2-L3 (all except H) and MW2-L1&L2 (H): (A), (B), (C), (D), and (F) are ‘classic’ hammerstones from MW2-L3; (E), (G), and (H) are hammerstones with fractured angles. All except (C) are made on basalt. (C) is made on glassy ignimbrite.

Because identification of the range of percussion activities is a difficult and often subjective task, the typo-technological criteria used to recognize hammerstones have been variable throughout research history [39, 50, 104, 123, 135–137]. Here two types of hammerstones were identified, based on the characteristics of percussion traces and fractures on their surfaces. One type is the ‘classic’ hammerstone with battering marks and/or small pitting depressions on the cortical, often round, surfaces (Fig 11A, 11D and 11F). The other type is the hammerstone with fractured angles, in which battering is associated with fractures or scars that likely resulted from heavy blows during knapping (e.g., Fig 11E, 11G and 11H). The power loading during hard hammer knapping often leaves negative scars on the surface of hammers and, at times, results in splitting of the hammer itself and the formation of irregular ridges along elongated planes [136].

In both MW2-L3 and MW2-L1&L2 assemblages, basalt appears to have been the preferred raw material for use as hammerstones, followed by glassy ignimbrite and scoria (S1 Table). Unlike the pattern in the debitage and LCTs, ignimbrites are rare or absent among hammerstones in MW2 assemblages, suggesting an informed selection of basalt and glassy ignimbrite cobbles of various sizes to be used as hammerstones. Hammerstones from MW2-L3 are dominated by items with fractured angles (58.7%; *n* = 27), the remaining items being ‘classic’ hammerstones. The majority of hammerstones were made on round cobbles with only few of them on elongated items. The MW2-L3 hammerstones are on average heavier than cores in the assemblage (S4 Table; M-W test: U = 3662.5, *p < 0*.*0001*). On the contrary, cores are on average heavier than hammerstones in MW2-L1&L2, but the difference is not statistically significant (M-W test: U = 108, *p = 0*.*77*).

## 4. Discussion

Hominin occupations in the MW2 stratigraphic sequence are associated with flood plains or with channel beds. The archaeology-bearing layers were buried by sedimentation processes associated with fluvial systems (see section 1.2.; [1, 138]). In such contexts, the physical characteristics of artifacts are one of the means of evaluating the effects of formation processes and assessing the degree of assemblage integrity in order to contextualize typo-technological observations [139–141].

The composition of the MW2-L3 assemblage suggests channel dynamics may have caused winnowing and loss of some of the smaller and lighter fraction of lithic items [142–144]. Otherwise, taphonomic characteristics of the lithic items indicate only little post-discard disturbance or damage (S5 and S6 Tables). The accumulation of the artifacts and associated fauna thus appears to be due mainly to anthropogenic activities.

The lithic assemblage of MW2-L4 is too small and the context of MW2-L1&L2 too unclear to draw contextualized conclusions about depositional contexts. Still, given their relatively pristine preservation (in terms of abrasion and patination), artifacts from the latter context seem to have been exposed and dislocated only very recently, by sand quarrying activities. The low frequencies of abraded and patinated items supports the view that these items had been accumulated on the Early Pleistocene landscape as a result of human activities rather than by geogenic transport (S5 and S6 Tables), rendering these assemblages suitable for analyses of diachronic inter-assemblage variation.

### 4.1. Techno-economic characteristics of the MW2 sequence

Our analyses show that the lithic technologies of the various MW2 occupations are consistent with the Acheulian technocomplex. Patterns of raw material selection, procurement and transport, in combination with the technological attributes of the various components of the assemblage, confirm the co-existence of two distinct lithic technological systems (*chaînes opératoires*)–one for small to medium-sized flakes and another focused on the process of production of LCTs. This is manifested in the raw material economy as well as in the technological procedures applied.

#### Raw material procurement

Organizational decision-making entails weighing the influence of each of many factors that affect hominin survival in a given ecological context, with the aim of mitigating discrepancies in the spatial and temporal distributions of the various resources. Within such behavioral systems, the criteria for selection, transport and use of various raw materials are subservient to the availability of primary subsistence resources (e.g., [13, 59, 71, 121]). Lithic production processes in the two chronological phases represented at MW2 suggest informed decision-making with regards selection and procurement of lithic raw materials, attesting to behavorial flexibility with time.

Lithic production and use of the MW2 stone-tool makers relied on ignimbrite and glassy ignimbrite. Together, these raw materials constituted ≥80% of all artifact categories across assemblages (Tables 2, 5 and 7). The distributions of these rock types do not overlap on the modern landscape [115]. Today, extensive exposures of glassy ignimbrite are observed along the Kawa River banks (see S5 Fig) some 4–5 km west of the MW localities. The exposures along the Wabe River banks are predominantly of fractured, crystalline ignimbrite (Resom A. [Unpublished]). While currently invisible in the immediate vicinity of the MW2 locality, presumably buried under the overlaying sedimentary successions, ignimbrite flows are observed at other localities, stratigraphically equivalent with MW2-L4 and MW2-L3 (MW3, MW6, MW8; Fig 2; [1]). Giant cores and boulders associated with archaeological horizons at MW3 and MW8 suggest that ignimbrite flows were accessible to the Acheulian tool-makers and may have been utilized as a source for lithic raw material. The channel beds of the Gadeb plain’s streams may have served as additional sources of ignimbrite cobbles that were used to produce the small to medium-sized debitage.

Knappers of the artifacts in the two layers grouped by us as ‘MW2-L1&L2’ had access to the same raw material sources as their predecessors.

In MW2-L3, ignimbrite dominates the cores for small to medium-sized flake cores, large cores, and large flakes (Tables 2 and 5). This raw material likely was procured directly from bands of ignimbrite situated near the locality and from secondary sources in the network of stream channels. Glassy ignimbrite occurs in significant frequencies only in the châines opératoire for small debitage.

In both MW2-L3 and MW2-L1&L2 assemblages, the average size of glassy ignimbrite cores is significantly smaller than that of cores made on ignimbrite (Table 4; section 3.2.), suggesting that they were mostly made on cobbles transported fluvially downstream from the Kawa (or from [currently unknown] sources in the Bale Mountains). This is consistent also with the higher proportion of glassy ignimbrite (compared to ignimbrite; Table 6) among regular flakes. Pumiceous ignimbrite, used sporadically in the assemblages, occurs as isolated exposures in the upper sections (post-dating all archaeological layers) of MW2 type-locality. We are currently unaware of such exposures that are contemporaneous with the Acheulian occupations and assume that this ignimbrite type was also retrieved from the channels.

Size comparison between cores and their unmodified counterparts (i.e., natural items) from the *in situ* MW2-L3 manifested non-random selection pattern (see section 3.2.), indicating that knappers made informed selection of the sizes of raw materials from the locally available pool. Likewise, knappers of MW2-L3 and MW2-L1&L2 made informed decision of exploiting mainly denser and heavier basalt (compared with ignimbrites; see section 3.5.) for utilization as hammerstones. Their larger mass, enabling the effective loading of the force needed in the knapping activities, could have been one of the main reasons behind their preference as hammerstones.

#### Small debitage techno-economy

MW2-L4 and MW2-L3 occupants focused on the production of small to medium-sized flakes from cobbles/pebbles of varied sizes, employing various technological strategies (Table 5; S7C & S7D Fig). Elements associated with LCT production constitute a minor proportion of the reduction sequence (~0.2% of the entire assemblage; Table 1; S7 Fig).

Raw material exploitation pattern manifested significant variability over time. In MW2-L3, marked contrast was observed between the raw material composition of small debitage assemblage where ignimbrite dominated the core assemblage while glassy ignimbrite dominated the debitage categories, except large flakes (see Table 2). The contrast between the two components of the assemblage suggests differences in raw material economy, with preferential exploitation of glassy ignimbrite for the production of small to medium-sized debitage. The differential treatment of raw materials seen in MW2-L3 appears to have transitioned into a preferential exploitation of single raw material (i.e., glassy ignimbrite) in the context of MW2-L1&L2. In addition to LCTs, cores and large flakes were also dominantly made on glassy ignimbrite (see Tables 2 and 5). This suggests a diachronic shift towards the exploitation of raw material with superior knapping properties (i.e., relatively aphanitic and homogenous; see S1 Text).

MW2 knappers practiced elaborate core reduction strategies that suggest increasing efficiency of exploitation over time. Bifacial knapping techniques were preferentially employed in both MW2 cores assemblages. In MW2-L3, those cores show mainly peripheral exploitation indicative of shorter core lives, whereas strong attention was given to the maintenance of knapping surfaces in the younger MW2-L1&L2. In addition to extending core use-lives, the increasing frequency over time of cores associated with structured knapping techniques indicates more investment in maintenance and is indicative of higher efficiency of knappers’ technical skills. For cores with reduction restricted to a single surface, knappers followed a strategy of extending the exploitation sequence from the onset, with increasing intensity of exploitation in the younger MW2-L1&L2 assemblage.

The intensity of core exploitation supports the trends observed from the analysis of core reduction scheme. Compared with MW2-L3 cores, there is a marked increase in the average scar number on MW2-L1&L2 cores and the diachronic increase is skewed towards glassy ignimbrite (see section 3.3. and Fig 5). The diachronic shift towards a preferential use of glassy ignimbrite thus appears to have been accompanied with an increase in knapping intensity aimed at maximizing the productivity.

Regular flakes dominate the small to medium-sized flakes produced in the MW2-L3 assemblage (S7A & S7B Fig). These flake types were also rarely transformed by secondary modification into formal tools. Of four such instances, three are simple side-scrapers, while one is an atypical end-scraper. Thus, the cutting-edge(s) of ‘ordinary’ flakes may have been directly utilized when needed.

#### Large Cutting Tool technologies and reduction sequence

The utilization of at least three distinct flaking methods for the production of LCTs blanks was observed in both MW2 assemblages. Yet, these methods were variably employed with the transversal flaking methods preferred over other methods in both assemblages (see section 3.4.). This pattern suggests that MW2 Acheulian knappers in both occupation phases utilized cost-effective knapping strategies from the outset. In terms of raw material selection, glassy ignimbrite was preferred over any other rock type for LCTs production in both MW2 assemblages (Table 7). Thus, MW2 hominins followed a strict protocol of raw material selection for the production of LCTs.

Importantly, the dimensions of large flakes as well as of dominant scars on large cores in MW2-L3 are smaller than flake-based massive crude LCTs in the same assemblage (Table 9; S2 Table). Combined with the fact that most of those massive items were made on blanks of glassy ignimbrite (minimally represented in the large flakes and large cores), it is plausible that the blanks of the crude LCTs were detached off-site and transported into MW2-L3. Likewise, large flakes in MW2-L1&L2 are found to be significantly smaller than the handaxes and picks in the assemblage (see section 3.4.). This discrepancy is independent of any size-related bias during collection, and therefore reflects off-site procurement and production of blanks for LCTs, similar to MW2-L3.

Consistent with the patterns of raw material of cores and LCTs, flakes associated with the *façonnage* of bifacial tools are predominantly of glassy ignimbrite, while those related to core modification are mostly of ignimbrite (Table 6). In MW2-L3, the high frequency of thinning flakes among the *façonnage* flakes and the low frequencies of roughing-out flakes (Table 6) suggest that technological activities on-site focused on late stage shaping of bifacial tools (see section below). Associating the relative proportion of *façonnage* flakes in the MW2-L1&L2 assemblage is difficult at this stage due mainly to the probability of size-related collection bias.

The number of LCTs and cores in the MW2-L1&L2 assemblage is nearly equivalent (compared with only 0.2% of LCTs in MW2-L3; Table 1). Combined with the marked increase in the proportion of LCT shaping flakes (see Table 6), which cannot be explained solely by collection bias, this seems to be related with higher emphasis on the production of this artifact class. This may be indicative of a shift in the goal of *châine opératoire* towards emphasizing the production of LCTs in MW2-L1&L2. (A similar shift is also observed in age-equivalent *in situ* assemblages of locality MW5; see Table 5 in [1]; manuscript under preparation).

The higher emphasis placed on the production of LCTs in the ~1.4 Ma MW2-L1&L2 assemblage appears to have been accompanied by a shift in raw material economy. The preferential exploitation of glassy ignimbrite from a relatively distant sources would require higher investment of time and energy to exploit those sources. Under such conditions, the strict selection of raw materials caused spatial and temporal fragmentation of reduction sequences, where different stages of the process took place in different places on the landscape. The fragmentation of reduction sequence observed at the MW site-complex around 1.4 Ma, driven by the need for higher quality of raw material, suggests that this strategy of raw material economy started on the highlands much earlier than previously thought ([13, 48]; see also section 4.3. below).

### 4.2. Temporal trends in the shaping processes of Large Cutting Tools at MW2

The MW2-L3 LCT component is characterized as an incipient Acheulian technology with only very few crude LCTs, picks and large scrapers. Typical bifacial tools are absent from the assemblage, although large flakes were produced (0.84% of the debitage; Table 2). The majority of large flakes are side-struck, but end-struck and special side-struck blanks were also used. Thus, the flexible use of three distinct flaking methods for the production of large flakes is seen at MW2 at ~1.6 Ma.

The successfully detached large flakes retained their massive volume, even after the removal of few deep scars from the perimeter of the large blanks. These removals may have been unsuccessful attempts to further reduce or shape the large blanks, resulting in their early discard with an overall crude aspect (Fig 8A–8C). When the knappers succeeded in detaching large flakes with a thinner profile (denoting manageable volume, Fig 8E and 8F), they were not converted into the ‘typical’ bifacial tools (handaxes or cleavers); instead, they were retouched into large scrapers or utilized as knives.

The presence of large cores and the crude nature of bifaces in MW2-L3 suggests at least some on-site reduction of these items and leads to the expectation that roughing-out will occur in the assemblage along with a relatively low number of thinning flakes. While the proportion of LCT shaping flakes is relatively high (see Table 6), thinning flakes outnumber roughing-out flakes (Table 6; S3 Fig). Two alternative scenarios are possible. First, because massive flakes from this archaeological horizon are voluminous, roughing-out removals may have failed to fully reduce mass from the original large blanks, resulting in relatively smaller flakes. As the distinction between roughing-out and thinning flakes is somewhat blurred, being similar in most attributes except size (see S1 Text), the under-representation of roughing-out flakes may stem from a classification error. An alternative scenario is that the technological repertoire used by MW2-L3 knappers was restricted to rudimentary bifacial shaping procedures. In this case, the low frequency of finishing flakes represents a more restricted control of the knapper over the technological means of standardizing bilateral edges and bifacial profiles of bifaces.

At MW2-L1&L2, the higher proportion of roughing-out flakes (Table 6) suggests on-site execution of the early phases of bifacial production. The detachment of LCT blanks was coupled with shaping process resulting in more ‘typical’ handaxes, some picks, and rarely cleavers and large scrapers (Table 8; Figs 9 and 10). A large Kombewa flake also made the first (albeit isolated) appearance in this assemblage in the MW2 sequence but was not shaped into any LCT (S6 Fig). The poor representation of thinning and finishing flakes can be partly attributed to collection bias, which would artificially increase the frequencies of the larger, more visible roughing-out flakes. Still, the scars observed on the surfaces of bifaces are deeper and bigger, consistent with technological practices that would have led to a lower number of finishing flakes.

The volumetric configuration of the bifaces themselves indicates relatively low investment in the standardization of the biface volume (Figs 9 and 10), either because they were sufficient for a perceived task or because the skill levels of the knappers would not allow more refined shaping. It is an aspect of LCT production that is similar in the MW2 assemblages at ~1.6 Ma and at ~1.4–1.3 Ma.

### 4.3. MW2 and the early Acheulian in the eastern African context

The earliest appearance of the Acheulian lithic technology has been documented in the Rift Valley sites at Kokiselei (KS4, [19]) and Konso (KGA6-A1, [20]) at around 1.75 Ma years and in Oldupai Gorge (FLK-West, [21]) a little later at around 1.7 Ma. By ~1.6 Ma, early Acheulian assemblages were described at Konso (KGA4-A2), Gona (OGS-12, [145, 146]) and at Melka Kunture (Garba IVD and Gombore IB, [17, 44]) on the highland. The assemblages from MW2-L4 and MW2-L3 therefore represent two additional and broadly contemporaneous instances of the poorly documented presence of early Acheulian hominin and their lithic technology at the high-altitude environments outside the Main Ethiopian Rift. Thus, by 1.6 Ma, the early Acheulian technology had spread into varied paleoecological, paleoclimatic, and paleogeographic environments (Fig 1; [1], Gossa T. [Unpublished]). This pattern is consistent with the ecological plasticity of the later phase of the Oldowan technocomplex (<1.8 Ma), known from Africa as well as Eurasia.

The technologies of lithic assemblages in the Melka Wakena and Melka Kunture 1.6 Ma-old assemblages bear strong similarities. In all the reported assemblages, the goals of the reduction sequence focused primarily on the production of small debitage (cf. [17, 44]) and LCT technologies were of incipient nature, such that large flake blanks preserved massive profiles (described by Gallotti [44:22] as of “great thickness”) that were rarely transformed by shaping. “Large cutting tools at Garba IVD can be considered as massive scrapers, …., in which the retouching never aims to manage the whole volume of the object or divide it into two different planes” ([44]:23). Likewise, the Acheulian technology in the KGA4-A2 and OGS-12 assemblages is also of incipient nature. Still, the targets of reduction and *façonnage* processes appear to be divergent, where KGA4-A2 and OGS-12 toolmakers manufactured four distinct types of LCTs, all massive—crude LCTs, picks, cleavers, and large scrapers [20, 148]. It seems that by 1.6 Ma the techno-economic rationales underlying a growing emphasis on LCT production were well underway in the Konso site-complex (and probably OGS-12 of Gona), while hominins that occupied the highland environments (MW2-L4 and MW2-L3, Garba IVD and Gombore IB of Melka Kunture) mainly relied on small debitage technology with only the occasional production and use crude LCTs elements. Whether this distinction is related to differences in paleoecological settings and resource structure, resulting in differences in exploitation pattern, to the taxonomic affiliation of the toolmakers, or any other factors, remains an open question for future exploration.

At around 1.4 Ma, repeated early Acheulian occupations have been documented at Konso (KGA10, KGA8, KGA7-A1&A3, KGA7-A2; [20, 147]), sites in Koobi Fora eastern Turkana basin [51], localities EF-HR [46], TK [49, 94], and BK [50] in Oldupai Gorge, some localities of Peninj site-complex [21, 47, 148], and Melka Wakena (MW2-L1&L2, MW5-L1 and MW5-L2; [1], Gossa T. [Unpublished]). The majority of assemblages from those sites demonstrate an early yet full-fledged Acheulian technology that had shifted to more frequent and more elaborate production and use of typical handaxes, picks, cleavers, and large scrapers. In some cases (e.g., Gombore Iγ and Iδ at Melka Kunture [1.4–1.3 Ma; Mussi et al., 2021] and BK4b site in Oldupai Gorge [50], however, knapping was dominated by reduction sequences dedicated to small debitage production, as in the earlier assemblages. At the end of early Acheulian, fragmentation of LCT reduction sequence has been documented in some sites, such as, Garba XIII (dated 1.0–0.8 Ma) and Gombore II (0.8–0.7 Ma) of the Melka Kunture site-complex ([13, 48] and references therein to dating and stratigraphy). At MW2-L1&L2, this behavior is detected at around 1.4 Ma, which may imply differences in the tempo of behavioral adaptations among the highland occupations themselves.

Overall, the patterns of lithic production in 1.4 Ma assemblages in both the rift and the adjacent highlands underline the widespread distribution—geographically and ecologically—of early Acheulian hominins in eastern Africa at that time, congruent with Clark’s (1987) model of large-scale expansion of the range of habitats exploited by *Homo erectus* in second half of the Early Pleistocene.

## 5. Conclusions

The Melka Wakena and Gadeb early Acheulian site-complexes, as well as the Melka Kunture sites, are among the very few sites that record hominin habitation of the highland environments outside the Rift System during the Early Pleistocene. Occupation horizons at MW2 represent one of the earliest known arrivals of Acheulian hominins to the highlands and their prolonged, albeit intermittent, presence at this locality (~1.6 Ma–~1.3 Ma). Occupants of this locality were equipped with the early Acheulian technology characterized by the co-existence of lithic châines opératoires for small debitage and for LCT production, respectively. The technocomplex is characterized by dynamic reduction sequences with elaborate raw material economy and technological changes with time. MW2 knappers employed complex core reduction strategies that manifested increasing efficiency of exploitation and advancement of the knappers’ technical skills with time. The reduction sequence reliant on the production of small debitage in the earliest assemblages of MW2-L4 and MW2-L3 (~1.6 Ma) shifted towards the reduction sequence oriented towards LCT production in the context of MW2-L1&L2 (~1.4 Ma). A generalist, diverse raw material exploitation in the production of MW2-L3 small debitage transitioned into preferential exploitation of a highly knappable glassy ignimbrite in MW2-L1&L2. This seems to have been coupled with increasing intensity of knapping this raw material, aimed at maximizing productivity. This shift in raw material economy may have occurred at MW2 much earlier than previously reported from elsewhere on the highland (e.g., [48]), documenting for the first time coeval inter-assemblage variation in the pace of behavioral adaptation in this habitat.

MW2 hominins preferred glassy ignimbrite and flake blanks for LCT production from the onset, a behavior that remained conservative in the ~300 kyr represented by the locality’s record. The emphasis on exploitation of quality raw material for LCTs is indicated in the off-site production of large flakes and their transport into the locality, resulting in spatiotemporal fragmentation of reduction sequence. At the same time, the technological behavior associated with the production of the flake blanks shows high flexibility from its early phases, when knappers employed various cost-effective strategies to successfully detach blanks from boulder/giant cores. At ~1.6 Ma, the detached blanks retained massive volumes that the knappers found challenging for further shaping during the façonnage stage. These items were discarded as crude LCTs. The debitage technology of LCTs shows refinement in the context of MW2-L1&L2 (~1.4 Ma), when knappers often produced blanks with manageable volumes that they later shaped into typical Acheulian LCTs (handaxes, picks, a cleaver, and large scrapers). The refinement of LCTs technologies with time, observed at the MW2 early Acheulian assemblages, echoes the overall trends of evolution of this technocomplex, previously noted in early Acheulian sites such as Konso and Melka Kunture. Beyond documenting the technological characteristics of new assemblages and expanding the database for the early Acheulian in general, this study reveals the internal dynamics of changes and continuities in the early Acheulian techno-economy of the highland, demonstrating that it tracks technological developments within the Rift Valley at a relatively short time lag. The similarity in production systems across the two habitats, despite the differences in environmental background, speak to the flexibility of the technological behaviors of early Acheulian hominins when facing variable ecological conditions. These findings may have implications for understanding the broader dynamics of hominin movement within diverse geographic and ecological regions of eastern Africa as well as their expansions to more distant regions within and outside the continent.

## Supporting information

S1 File (DOCX)

S1 Text Contains the supporting texts for this manuscript [petrographic analysis; production of large flake blanks; core reduction scheme; characterstics of large cutting tool shaping flakes]. (DOCX)

S1 Fig Schematic description and actual example of the (A) transversal, (B) the oblique, and (C) longitudinal flaking techniques as expressed on giant cores (MW6).
(TIF)

S2 Fig Hypothetical scheme of free-hand core reduction followed in this study (from [20]; see also [21, 22]. (TIF)

S3 Fig LCTs Shaping (*faconnage*) flakes from MW2-L3 and MW2-L1&L2. (TIF)

S4 Fig Position and measurement protocols of (A) cores, (B) whole flakes, (C) handaxes, and (D) cleavers.
(TIF)

S5 Fig Location of modern-day exposures of glassy ignimbrite (left) and strongly welded tuff (ignimbrite) (right) in the vicinity of the MW localities.
(TIF)

S6 Fig Large Kombewa flake retrieved from MW2-L1&L2 designated collection area. (TIF)

S7 Fig Scatterplot showing size (max. length by max. width) distribution of (A) all flakes from MW2-L3, (C) MW2-L3 cores, and (D) MW2-L1&L2 cores. (B) Proportional representation of various flake size categories of MW2-L3 flake assemblage.
(TIF)

S1 Table Absolute and relative frequencies of percussive items and natural and indeterminate clasts (per raw materials) of MW2 assemblages. (DOCX)

S2 Table Dimensions of large flakes from MW2 assemblages. (DOCX)

S3 Table Descriptive statistics of the frequency of scars (removals) on the dorsal and ventral faces of large cutting tool assemblages. (DOCX)

S4 Table Descriptive statistics of core and hammerstone weights. (DOCX)

S5 Table Physical properties of core assemblages from MW2 occupation layers. (DOCX)

S6 Table Physical properties of LCTs from MW2 occupation layers. (DOCX)
