# Supplementary material for: Continuity and change in lithic techno-economy of the early Acheulian on the Ethiopian highland: A case study from locality MW2; the Melka Wakena site-complex
Source: PLoS One. 2022 Dec 7;17(12):e0277029. doi: 10.1371/journal.pone.0277029 (PMC9728887; doi:10.1371/journal.pone.0277029)
Supplement: S1 Text — (DOCX) [file pone.0277029.s002.docx]

**Supplementary Information (S1)**

**Petrographic analysis**

Glassy ignimbrite: Widespread exposures of flows of this rock are common along the banks of the Kawa River ([1], Resom A. [unpublished]). The rock presents a color gradient from light to dark green. In thin section it is porphyritic with big phenocrysts of orthoclase within a glassy matrix and minor quartz crystals. The phenocrysts are <1mm up to 8mm big, euhedral to sub-hedral, while the fiammes and rock fragments show a general elongation and flattening along the direction of welding. In addition, the orthoclase phenocrysts themselves contain minor older, accessory minerals forming poikilitic texture. Some accessories include alkali pyroxenes, and opaques (possibly ilmenite and hematite) (see [1] Appendix II for details of mineral composition of glassy ignimbrite). The preferred orientation and elongation of the fiammes and the structurally deformed flow banding is typical of a pyroclastic flow that welded during rapid cooling. This ignimbrite produces sharp edges upon breakage, which is an outcome of a higher degree of welding associated with the rapid cooling process.

Crystalline (‘regular’) ignimbrites: This rock is found mostly as exposed flows along the Wabe River Banks. It exists as fractured flow bands in locality MW6, whereas it is found as strongly fractured, weathered, sub-rounded to sub-angular blocks (10-70 cm in length) in localities of MW9, MW3, and MW7 (see Fig. 2 in the main text). Crystalline Ignimbrites sampled from those localities appear to differ in color and texture at hand specimen scale, but their mineral constituents and modal composition are similar (see Appendix II of [1]). In thin section they show a porphyritic texture with fine-grained (<1mm to 8mm long and <1mm to 2mm wide), oscillatory zoned, euhedral to subhedral orthoclase (15-20%). Fine-grained anhedral quartz (5-10%) phenocrysts are also set in fine-grained glassy matrix (65-70%). The matrix also contains fine alkali feldspar and quartz grains, with evident preferred orientation (flow banding) across the bedding direction. It contains rare fiammes (<5%). There are also fine-grained (<1mm-4mm long), irregular to sub-rounded and occasionally elongated, non-deformed but slightly altered lithic fragments (<3%). Some sanidine crystals are also observed. This ignimbrite produces sharp edges upon breakage, but manifests characteristics of lesser degree of welding compared with glassy ignimbrite ([1], Resom A. [Unpublished]).

Moderately welded (‘pumiceous ‘) ignimbrite: it is exposed in localities along the upper stream sector of the MW site complex. It is rarely observed in the downstream area of the site complex. This ignimbrite flow unit is seen today as an isolated exposure in the upper sections of the stratigraphic sequence in locality MW2, post-dating the archaeological horizons. It is fine-grained and contains slightly vesiculated pumice clasts. This mostly gray colored rock shows a moderate degree of welding (i.e., it is lighter compared with the two other ignimbrite types) and easily crumbles upon breakage.

**Production of large flake blanks**

The production of large flake blanks is perceived as a marker for the onset of the Acheulian technocomplex [2-5]. In the flake-based early Acheulian technology, procedures of *façonnage* used for shaping of ‘classic’ LCT are rare [6,7]. The morphology of the blank is determined to a large degree during the debitage stage (detachment of large flake blanks), which is therefore key to understanding the morphological goals, the processes, and the level of technical skill applied. The flaking orientation of the large flakes for LCT blanks provides direct clues as to the specific technical procedures employed to exploit the convexities on the surfaces of giant/boulder cores and to infer the knapping strategy.

Minimally three types of large flakes were identified in the MW2 assemblages: side-struck, end-struck, and special side-struck (corner) flakes. The division is based on the position of the striking platform in relation to the long axis of the piece. Following Isaac and Keller [16], the technological length of end-struck flakes is equal or longer than that of the width of the flake; side-struck flakes are those where the width equals or surpasses the length; special side-struck flakes are those where the longest dimension is a diagonal ‘chord’ from the bulb of percussion to the most distant part of the flake. These types are here associated with three modes of reducing giant/large cores.

Detaching large flakes from giant/boulder cores requires considerable force. While one may hypothesize that Acheulian hominins were physically capable of generating the necessary force, the detachment of large flakes could not be achieved without the knowledge and the practical skills that allowed them to successfully manipulate and exploit the geometries of giant/boulder cores [8-11]. The successful detachment of the LCT blanks is contingent on careful planning and precision [12].

At the MW site-complex, massive boulder cores (likely weighing 50-100 kg) are seen partially exposed to the surface in three different localities (MW3, MW6, and MW9) along Wabe River [17] and are under study. Regardless, our preliminary field observations show that exploitation patterns of those boulder cores related to detachment of large flake blanks are in agreement with flaking directions observed on the LCTs. We use material from these localities to visualize the reduction process of large cores.

Effective exploitation of giant/boulder cores starts with identifying suitable angles that would create practical striking platforms and allow the application of appropriate force to knock off large flake blanks [9,12,13]. From the *châine opératoire* point of view, the knapper is required to mesh abstract knowledge and practical know-how. Once boulders with suitable angles were selected, an effective knapper would identify the most suitable point of strike, adjust the force required for removing a desired end-product, and successfully apply this force [12].

***Transversal flaking*** is a cost-effective strategy for detaching large flake blanks that optimizes energy expenditure and influences the size and morphology of the end-products. It involves targeting the edge along the side of a boulder to knock off a large flake, with its longest axis perpendicular to the axis of flaking (Figure S1A). As the force radiates from the point of percussion in all directions, a wide (in plan view) and large flake can be detached by applying optimum force (see [14,15]. This method was employed frequently by MW hominins from the earliest periods represented in the site’s record (Table 6).

***Oblique flaking*** is an alternative strategy whereby special side-struck blanks (at times known as corner flakes; [16]) are detached from giant/boulder cores. The procedure is similar to that of transversal flake removal, the difference being that the intersection of two adjacent striking platforms (i.e., “corner”) is used as a point of hammer impact rather than a point on the side of a boulder (Figure S1B). The direction in which force radiates is oriented obliquely to the longest axis of the resultant flake. Madsen and Goren-Inbar [8] suggest that those flakes were produced by using ‘the scars of a previous flake as the striking platform for the detachment of the next flake’ (see also [9, 10]).

***Longitudinal flaking*** is a third approach to obtaining large flakes, in which the flake’s longest axis is oriented in the direction of blow (‘end-struck flakes’; Figure S1C). Compared to transversal flaking, this technique requires application of greater force, as energy needs to penetrate deep inside the volume of the core in order for a large flake to spall off. This also makes this technique more susceptible to the effects of raw material texture (e.g., phenocryst size, matrix homogeneity) and may result in higher frequencies of hinge terminations or snapping of the intended flake [9,12].

Field surveys ([1], Resom A. [Unpublished]) indicate that the majority of boulders from ignimbrite flows within the MW study area, as well as the closest flows of glassy ignimbrite in the Kawa River area (4-5 kms away from MW2), are of angular and sub-angular morphologies. The naturally occurring angles on ignimbrite boulders from MW3 and MW6 localities, stratigraphically older or coeval with MW2 (Hovers et al.[17], their Table 1 and Fig. 1), served as initial striking platforms for blank removals from very large boulders (Figure S1). Several methods of giant core exploitation were identified ([17], Gossa T. [Unpublished]). Based on the negative scars found on the boulder cores, the products appear consistent with the types of flakes identified as LCTs blanks in the MW2 assemblages.

**Core Reduction Scheme**

De la Torre’s [20] core reduction schemes consider three technological procedures (‘properties’), each of which can attain variable attribute states:

1. Interaction of knapping surfaces: Cores can be classified into bifacial and unifacial exploitation systems. Unifacial reduction pertains to situations where flaking is restricted to a single surface (reduction modes USP, UAU1, UAU2, UABI, UAUT, UP, and UC). Cores showing “frequent exchange of knapping surfaces” (BSP, BAP, BALP, BALT, BP, BHC and Discoid methods) belong in the bifacial exploitation systems ([18]:775).

2. Rotation of knapping surfaces*:* During reduction cores are likely to be rotated for better grip and better flaking angles but not necessarily to the same degree. It effectively corresponds to the length of exploitation series. This technological action distinguishes full rotation (UAUT, UABI, BALT, UP, BP, UC, BHC, and Discoid systems, of relatively extended flaking) versus restricted rotation of the exploitation surface (USP, BSP, UAU1, UAU2, and BALP, of shorter flaking sequences).

3. Organization of core volume/knapping surface: This aspect, identified as structured versus unstructured reduction sequences [18], pertains to the management of the central volume of core in the knapping process. Most of the reduction modes above show exploitation restricted to the peripheral areas of the cores and are less effective in managing the central volume of cores. Discoid and BHC are typical examples of well-structured and successful exploitation of the central volumes of the cores. Multifacial and Polyhedral exploitation systems categorize cores exhibiting unstructured but successful maintenance of flaking surfaces.

**Characteristics of large cutting tool shaping flakes**

Experimental and replication studies demonstrated that some flakes play important technological roles in the production sequences that are most associated with the Acheulian, namely, bifacial tool production (e.g., [10, 21-24]). Such experimental works have devised useful protocols with which characteristic elements of technological process can be identified and linked to Acheulian reduction procedures. The analysis in this section focuses on the technological traits of such flakes in the early Acheulian technology at MW, documenting the characteristics of the technological products and by-products of the various procedure.

Some of these flake types are associated with the process of core exploitation, pertaining to various stages of reduction and to methods of knapping, and their identification allows reconstruction of these reduction processes. Among these are shoulder flakes, wedge-like flakes, and core trimming flakes [8-10].

Following core reduction, some types of flakes are associated with the preparation of the bifaces themselves. Three flake types specifically associated with the shaping of bifacial tools have been identified and described for the MW and comparative assemblages. These can potentially be associated with the stages of bifacial tool production is given below:

1. Roughing-out flakes: The characteristics of this flake type are similar to Newcomer’s [21] definitions, the main difference being that in MW most of the bifacial tools are made on large flakes rather than cobbles. Overall, roughing-out flakes in MW tend to be thick at their proximal ends, with wide and plain (sometimes cortical) striking platforms. The angle between the butt and ventral surface is often obtuse. The flakes carry variable amounts of dorsal face cortex. These flake types likely represent first generation removals from large flake blanks in the process of crafting LCTs. Flakes in this category are similar to biface thinning flakes but are larger.

2. Thinning (or sharpening) flakes: Flakes in this category combine features of flake types described by Goren-Inbar and Sharon [22] as ‘short and thick’ flakes and by Newcomer [21] as ‘thinning and shaping’ flakes. They are “wide, short, and very thick at their proximal end and their thickness decreases sharply towards the distal end“ (Goren-Inbar and Sharon, [22]:121). As in the roughing-out flakes, the angle between their butt and ventral surface is often obtuse. Goren-Inbar and Sharon ([22]:121) surmised these flakes usually resulted from removals intended to “…create an appropriate striking platform” for the detachment of finishing flakes. It is important to note that from the several flake types they included in the ‘thinning and shaping’ category, we were able to objectively identify and quantify the presence of the ‘short and thick’ flakes.

3. Finishing flakes: These are small and thin flakes removed from the edges of the bifacial tools [21]. One of the features distinguishing these flakes from thinning flakes is that they “frequently carry remnants of the edges“ of the bifacial tools on their striking platforms and/or on the edges of proximal end of the dorsal surface ([21]:90; [24]).

**Supplementary References**

1. Resom A, Asrat A, Gossa T, Hovers E. Petrogenesis and depositional history of felsic pyroclastic rocks from the Melka Wakena archaeological site-complex in South central Ethiopia. Journal of African Earth Sciences 2018; 142: 93-111.
2. Isaac GL. Chronology and tempo of cultural change during the Pleistocene. In: Bishop W, Miller J, editors. Calibration of Human Evolution. Edinburgh: Scottish Academic Press; 1972. p. 381–430.
3. Isaac GL. Foundation stones. Early artefacts as indicators of activities and abilities. In: Bailey GN, Callow P, editors. Stone Age Prehistory. Studies in Memory of Charles McBurney. Cambridge: Cambridge University Press; 1986. p. 221–41.
4. Beyene Y, Katoh S, WoldeGabriel G, Hart WK, Uto K, Sudo M, et al. The characteristics and chronology of the earliest Acheulean at Konso, Ethiopia. Proceedings of the National Academy of Sciences 2013; 110: 1584-1591.
5. Gallotti R. An older origin for the Acheulean at Melka Kunture (Upper Awash, Ethiopia): techno-economic behaviours at Garba IVD. Journal of Human Evolution 2013; 65: 594-520.
6. de la Torre I, Mora R. Technological behaviour in the early Acheulean of EF-HR (Olduvai Gorge, Tanzania). Journal of Human Evolution 2018; 120: 329-377.
7. Gallotti R, Mussi M. Two Acheuleans, two humankinds: From 1.5 to 0.85 Ma at Melka Kunture (Upper Awash, Ethiopian highlands). Journal of Anthropological Sciences 2017; 95: 1–46.
8. Madsen B, Goren-Inbar N. Acheulian giant core technology and beyond: An archaeological and experimental case study. Eurasian Prehistory 2004; 2: 3–­52.
9. Goren-Inbar N, Grosman L, Sharon G. The technology and significance of the Acheulian giant cores of Gesher Benot Ya'aqov, Israel. Journal of Archaeological Science 2011; 38: 1901–1917.
10. Goren-Inbar N, Alperson-Afil N, Sharon G, Herzlinger G. The Acheulian Site of Gesher Benot Ya’aqov, Vol. IV: The Lithic Assemblages. Springer: Cham; 2018.
11. Stout D, Hecht E, Khreisheh N, Bradley B, Chaminade T. Cognitive demands of Lower Paleolithic toolmaking. PLoS ONE 2015; 10: 0121804.
12. Hiscock P. Learning in Lithic Landscapes: A Reconsideration of the Hominid ‘‘Toolmaking’’ Niche. Biological Theory 2014; 9: 27–41.
13. Sharon G. Acheulian Giant Core technology: a worldwide perspective. Current Anthropology 2009; 50(3): 335-367.
14. Petraglia M, LaPorta P, Paddayya K. The first Acheulian quarry in India: stone tool manufacture, biface morphology, and behaviors. Journal of Anthropological Research 1999; 55(1): 39-70.
15. Petraglia M, Shipton C, Paddayya K. Life and mind in the Acheulean. A case study from India. In: Gamble C, Porr M, editors. The Hominid Individual in Context. Archaeological Investigations of Lower and Middle Palaeolithic Landscapes, Locales and Artefacts. London: Routledge; 2005. p. 197-219
16. Isaac GL, Keller CM. Note on the proportional frequency of side-and end-struck flakes. South African Archeological Bulletin 1968; 23: 17-19.
17. Hovers E, Gossa T, Asrat A, Niespolo EM, Resom A, Renne PR, et al. The expansion of the Acheulian to the Southeastern Ethiopian Highlands: Insights from the new early Pleistocene site-complex of Melka Wakena. Quaternary Science Reviews 2021; 253: 106763.
18. de la Torre I. The Early Stone Age lithic assemblages of Gadeb (Ethiopia) and the Developed Oldowan/early Acheulean in East Africa. Journal of Human Evolution 2011; 60: 768–812.
19. de la Torre I. Technological strategies in the lower Pleistocene at Peninj (West of Lake Natron, Tanzania). In: Schick K, Toth N. editors. The Cutting Edge: New Approaches to the Archaeology of Human Origins. Bloomington: Stone Age Institute Press; 2009. p. 93–113.
20. de la Torre I, Mora R. Technological Strategies in the Lower Pleistocene at Olduvai Beds I and II. ERAUL 112. University of Liège Press: Liège; 2005.
21. Newcomer MH. Some quantitative experiments in handaxe manufacture. World Archaeol. 1971; 3: 85–94.
22. Goren-Inbar N, Sharon G. 2006. Invisible handaxes and visible Acheulian biface technology at Gesher Benot Ya‘aqov, Israel. In: Goren-Inba N, Sharon G. editors. Axe Age: Acheulian Tool-making from Quarry to Discard. London: Equinox; 2006. p. 111–135.
23. Jones PR. Experimental implement manufacture and use; a case study from Olduvai Gorge. Tanzania. Philosophical Transition of Royal Society B 292 1981;1057: 189-195.
24. Jones PR. Results of experimental work in relation to the stone industries of Olduvai Gorge. In: Leakey MD, Roe DA. Editors. Olduvai Gorge Volume 5: Excavations in Beds III, IV and the Masek Beds 1968–1971. Cambridge: University Press; 1994. pp. 254–298.
