# Supplementary figures and images for: Continuity and change in lithic techno-economy of the early Acheulian on the Ethiopian highland: A case study from locality MW2; the Melka Wakena site-complex
Source: PLoS One. 2022 Dec 7;17(12):e0277029. doi: 10.1371/journal.pone.0277029 (PMC9728887; doi:10.1371/journal.pone.0277029)

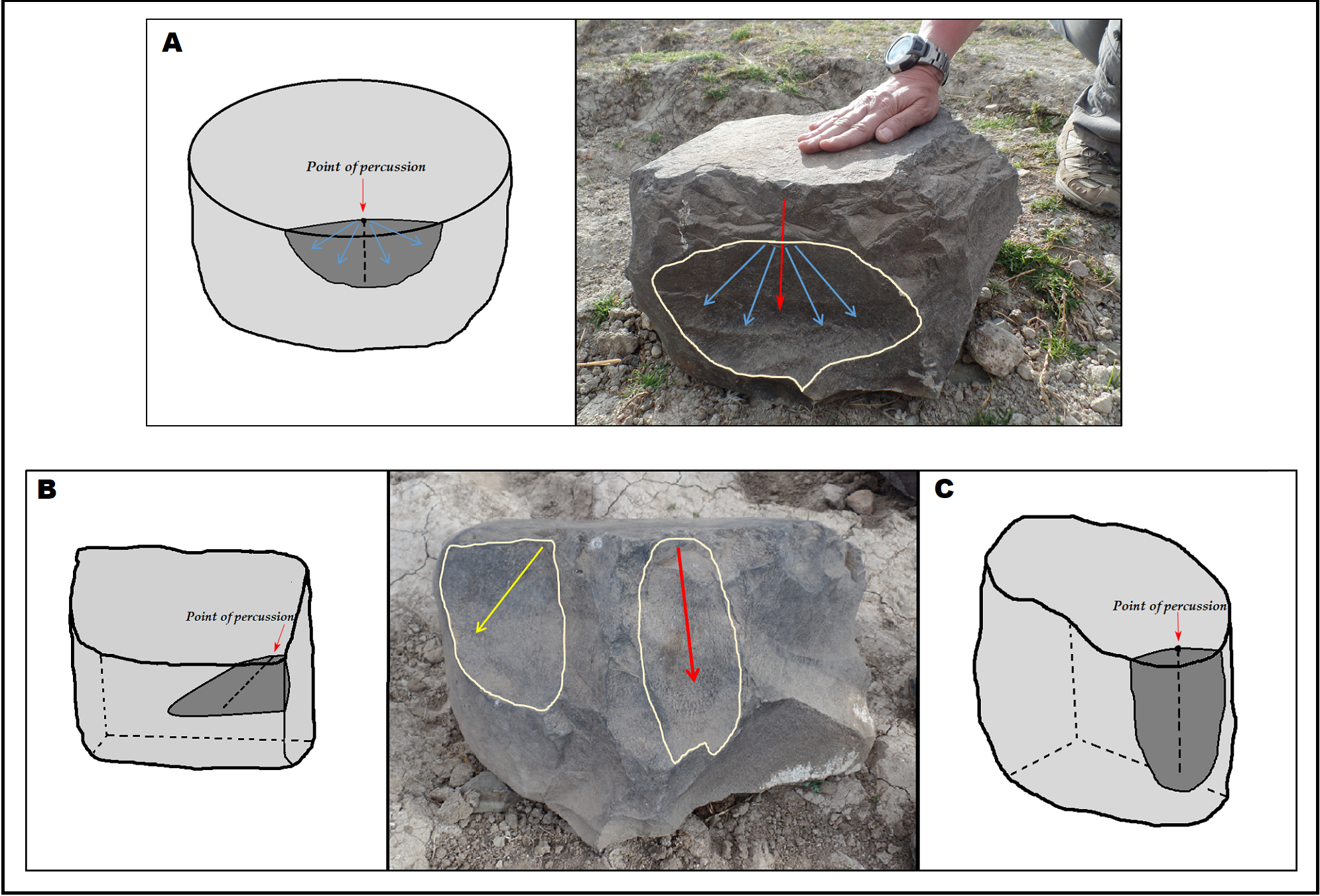

Supplement: S1 Fig — Schematic description and actual example of the (A) transversal, (B) the oblique, and (C) longitudinal flaking techniques as expressed on giant cores (MW6). (TIF) [file pone.0277029.s003.tif]

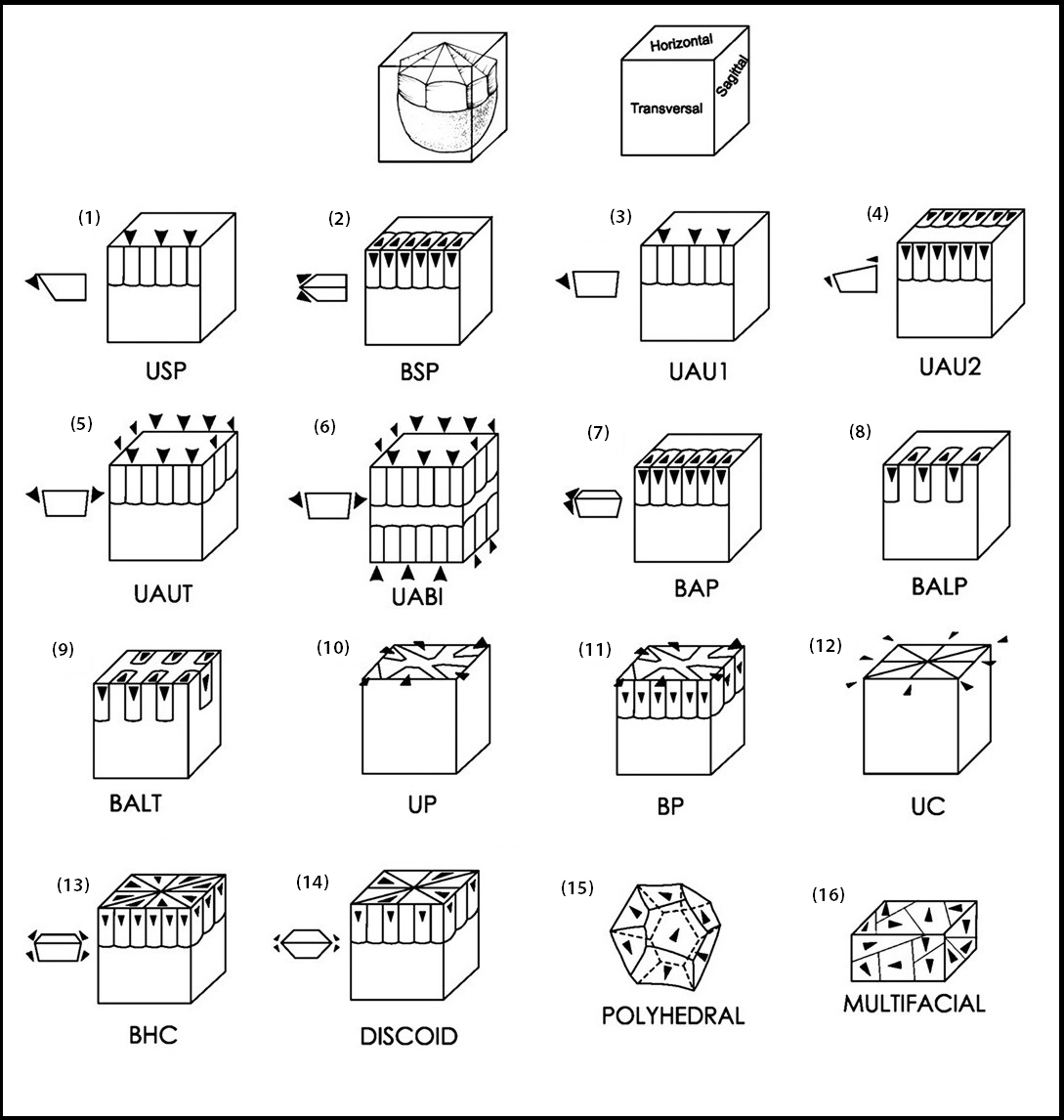

Supplement: S2 Fig — (TIF) [file pone.0277029.s004.tif]

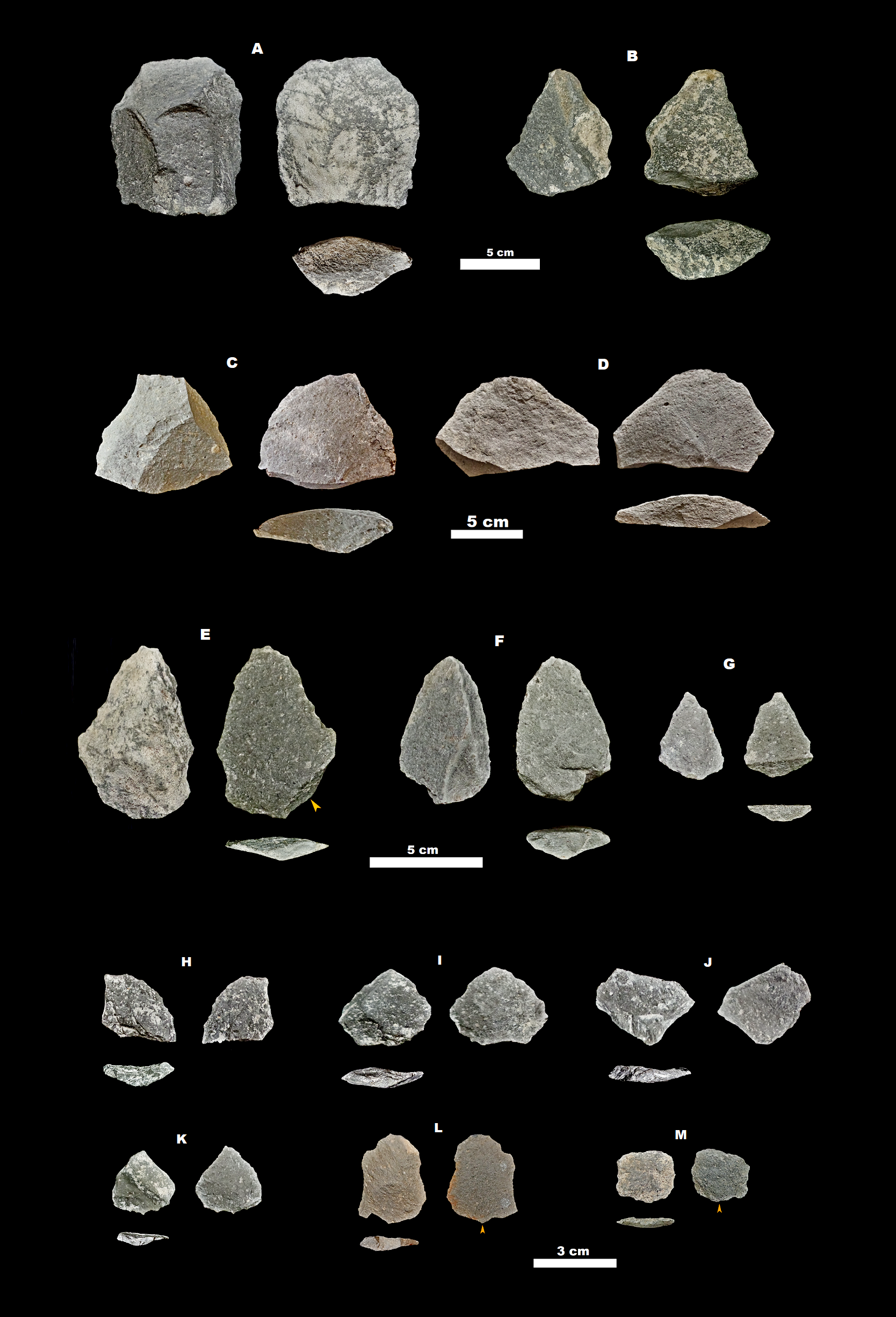

Supplement: S3 Fig — (TIF) [file pone.0277029.s005.tif]

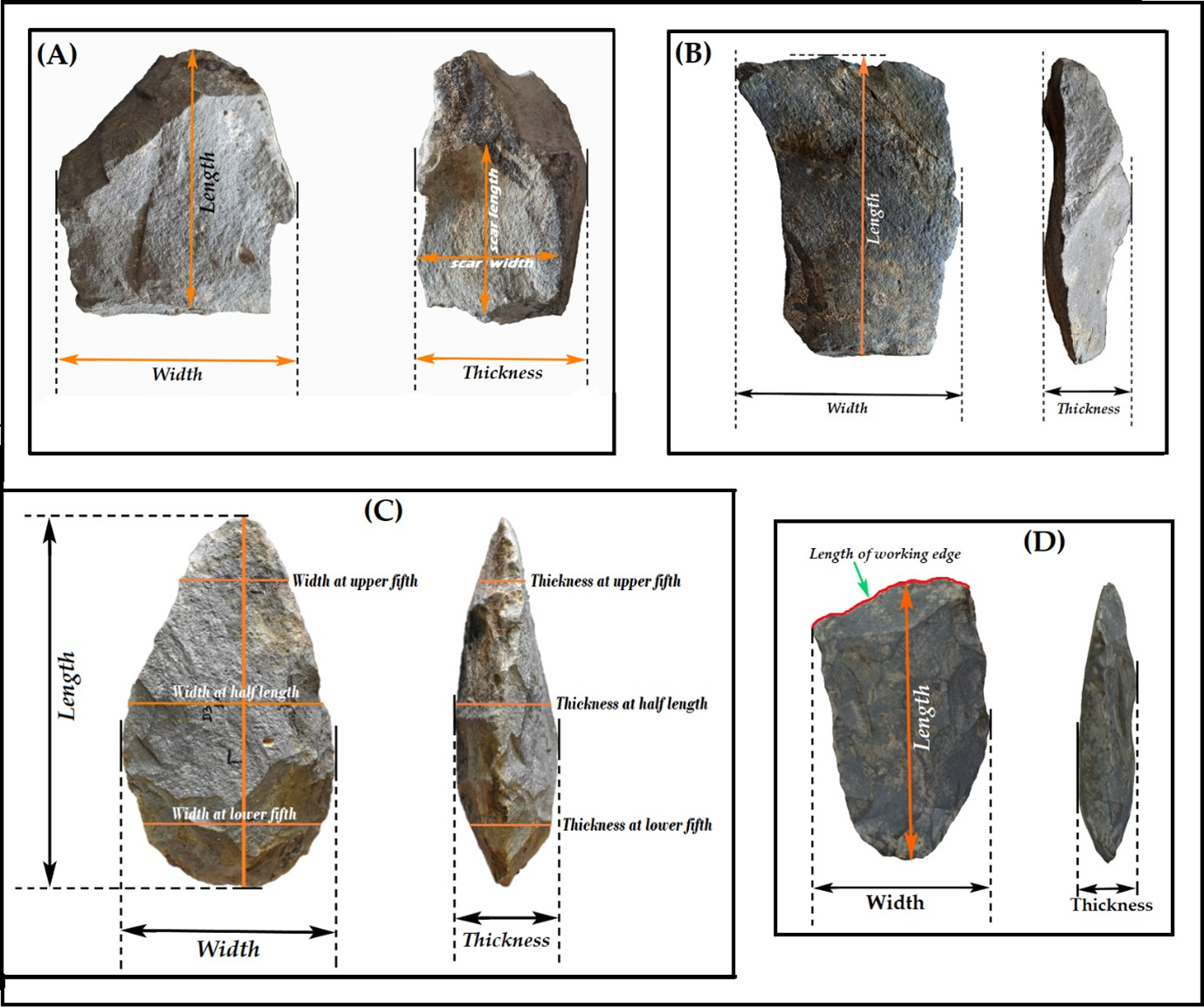

Supplement: S4 Fig — Position and measurement protocols of (A) cores, (B) whole flakes, (C) handaxes, and (D) cleavers. (TIF) [file pone.0277029.s006.tif]

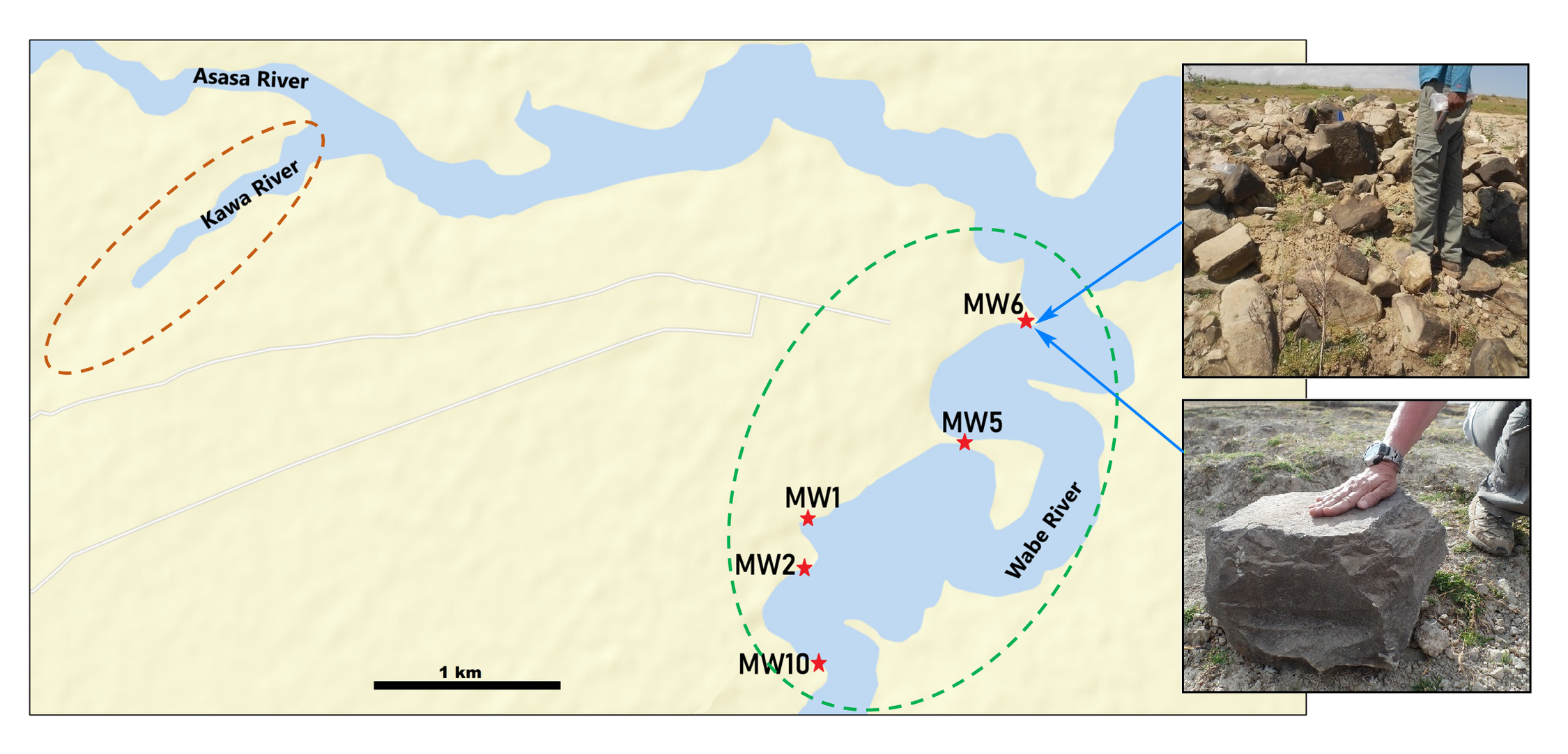

Supplement: S5 Fig — Location of modern-day exposures of glassy ignimbrite (left) and strongly welded tuff (ignimbrite) (right) in the vicinity of the MW localities. (TIF) [file pone.0277029.s007.tif]

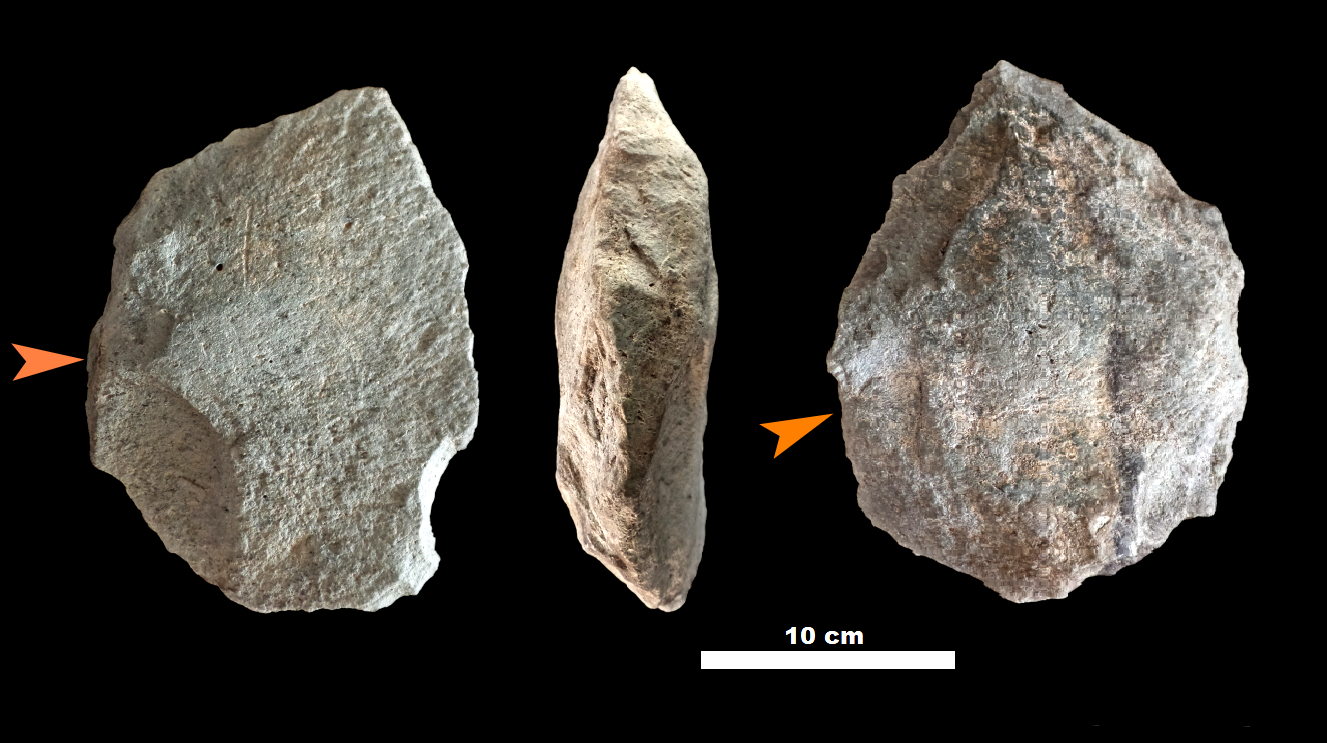

Supplement: S6 Fig — (TIF) [file pone.0277029.s008.tif]

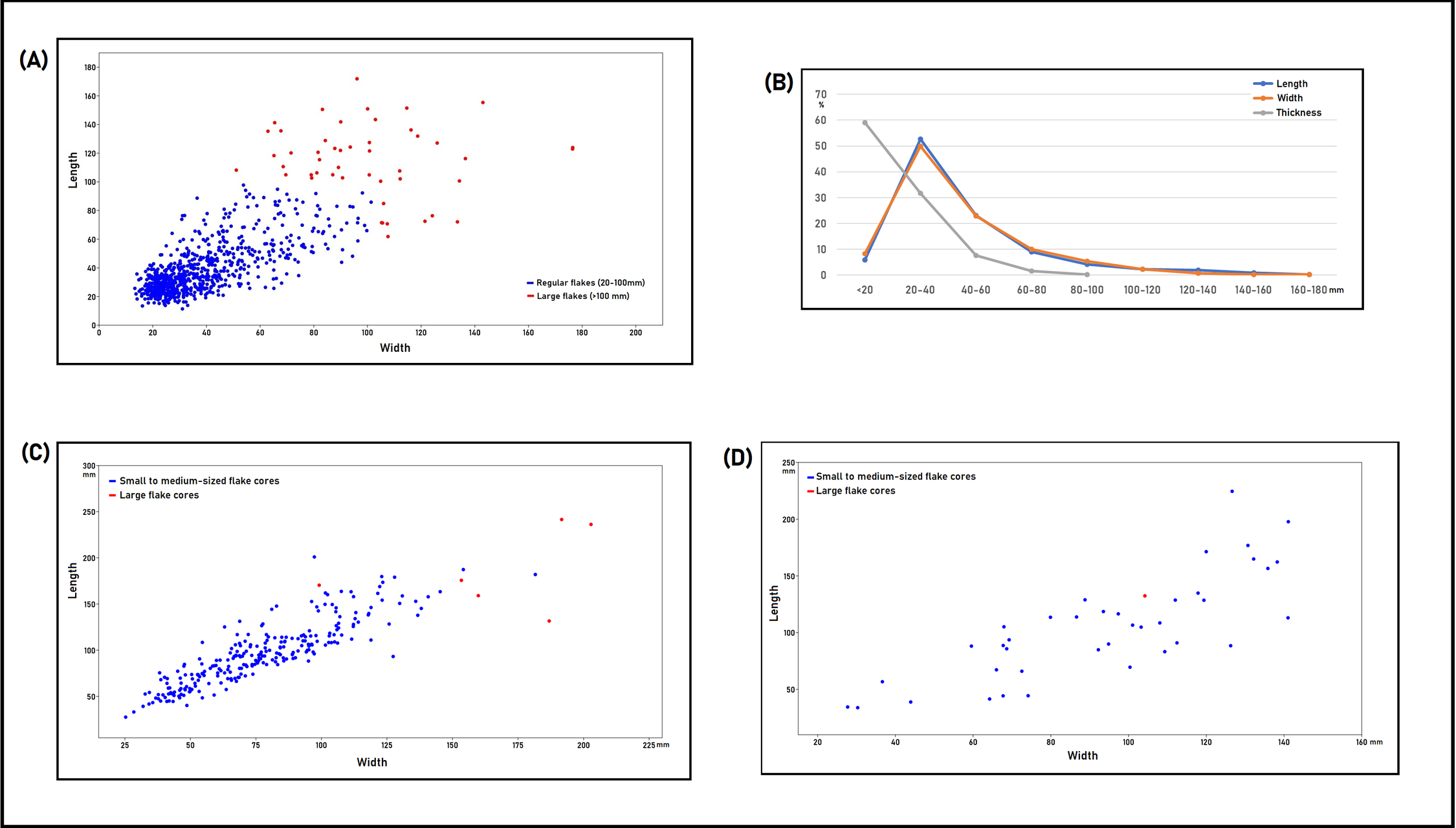

Supplement: S7 Fig — Scatterplot showing size (max. length by max. width) distribution of (A) all flakes from MW2-L3, (C) MW2-L3 cores, and (D) MW2-L1&L2 cores. (B) Proportional representation of various flake size categories of MW2-L3 flake assemblage. (TIF) [file pone.0277029.s009.tif]
