# Supplementary material for: Continuity and change in lithic techno-economy of the early Acheulian on the Ethiopian highland: A case study from locality MW2; the Melka Wakena site-complex
Source: PLoS One. 2022 Dec 7;17(12):e0277029. doi: 10.1371/journal.pone.0277029 (PMC9728887; doi:10.1371/journal.pone.0277029)
Supplement: S1 Table — (DOCX) [file pone.0277029.s010.docx]

**Table S1.1**

Absolute and relative frequencies of percussive items and natural and indeterminate clasts (per raw materials) of MW2 assemblages.

| Clast Category | *MW2-L3* | | | | | | | | | | | | | | |
| --- | --- | --- | --- | --- | --- | --- | --- | --- | --- | --- | --- | --- | --- | --- | --- |
|  | ***Glassy ign.*** | | ***Ignimbrite*** | | ***Pumiceous ign.*** | | ***Basalt*** | | ***Scoria*** | | | ***Other volcanic*** | | | ***Total*** |
|  | *n* | *%* | *n* | *%* | *n* | *%* | *n* | *%* | *n* | *%* | | *n* | | *%* | *n* |
| *Hammerstones* | **14** | 30.4 | **2** | 4.3 | **-** | - | **24** | 52.2 | **6** | 13.0 | | **-** | | - | **46** |
| *Natural items* | **4** | 3.63 | **59** | 53.6 | **29** | 26.4 | **7** | 6.4 | **10** | 9.1 | | **1** | | 0.9 | **110** |
| *Modified pieces* | **6** | 12.8 | **28** | 59.6 | **6** | 12.8 | **1** | 2.1 | **3** | 6.3 | | **3** | | 6.3 | **47** |
| *Indeterminates* | **85** | 14.8 | **319** | 55.7 | **9** | 1.6 | **42** | 7.3 | **-** | - | | **118** | | 20.6 | **573** |
|  | ***MW2-L1&L2*** | | | | | | | | | | | | | | |
| *Hammerstones* | **-** | *-* | **1** | 14.3 | **-** | - | **6** | 85.7 | **-** | | - | **-** | - | | **7** |
| *Modified pieces* | **-** | *-* | **-** | - | **2** | 100 | **-** | - | **-** | | - | **-** | - | | **2** |
