# Supplementary material for: Continuity and change in lithic techno-economy of the early Acheulian on the Ethiopian highland: A case study from locality MW2; the Melka Wakena site-complex
Source: PLoS One. 2022 Dec 7;17(12):e0277029. doi: 10.1371/journal.pone.0277029 (PMC9728887; doi:10.1371/journal.pone.0277029)
Supplement: S2 Table — (DOCX) [file pone.0277029.s011.docx]

**Table S1.2**
 Dimensions of large flakes from MW2 assemblages*.*

| Stat. | *MW2-L3* | | | | | | | | | | | |
| --- | --- | --- | --- | --- | --- | --- | --- | --- | --- | --- | --- | --- |
|  | ***Glassy ignimbrite*** | | | | ***Ignimbrite*** | | | | ***Pumiceous ignimbrite*** | | | |
|  | ***L*** | ***W*** | ***Th*** | ***Th/W*** | ***L*** | ***W*** | ***Th*** | ***Th/W*** | ***L*** | ***W*** | ***Th*** | ***Th/W*** |
| *n* | 4 | 4 | 4 | 4 | 34 | 34 | 34 | 34 | 10 | 10 | 10 | 10 |
| *Mean* | **118.7** | **87.8** | **50.6** | **0.583** | **115.7** | **98.5** | **51.1** | **0.544** | **115.8** | **110.4** | **52.9** | **0.492** |
| *S.D.* | 17.3 | 23.5 | 14.9 | 0.14 | 24.5 | 28.0 | 14.9 | 0.17 | 26.0 | 21.1 | 12.6 | 0.13 |
| *Min* | 102.8 | 62.9 | 32.5 | 0.411 | 70.7 | 51.1 | 24.1 | 0.203 | 76.3 | 81.1 | 36.3 | 0.342 |
| *Max* | 135.3 | 118.7 | 63.4 | 0.699 | 171.9 | 176.5 | 85.5 | 0.849 | 155.4 | 143.0 | 78.0 | 0.671 |
|  | ***MW2-L1&L2*** | | | | | | | | | | | |
| *n* | 11 | 11 | 11 | 11 | 9 | 9 | 9 | 9 | **-** | **-** | **-** | **-** |
| *Mean* | **123.8** | **105.8** | **54.8** | **0.545** | **149.3** | **120.8** | **51.9** | **0.446** | **-** | **-** | **-** | **-** |
| *S.D.* | 32.6 | 34.4 | 18.8 | 0.16 | 43.1 | 29.6 | 7.3 | 0.09 | **-** | **-** | **-** | **-** |
| *Min* | 75.8 | 66.9 | 35.0 | 0.296 | 99.3 | 89.8 | 39.0 | 0.313 | **-** | **-** | **-** | **-** |
| *Max* | 202.4 | 168.8 | 94.9 | 0.766 | 231.6 | 178.5 | 63.9 | 0.571 | **-** | **-** | **-** | **-** |
