# Supplementary material for: Continuity and change in lithic techno-economy of the early Acheulian on the Ethiopian highland: A case study from locality MW2; the Melka Wakena site-complex
Source: PLoS One. 2022 Dec 7;17(12):e0277029. doi: 10.1371/journal.pone.0277029 (PMC9728887; doi:10.1371/journal.pone.0277029)
Supplement: S3 Table — (DOCX) [file pone.0277029.s012.docx]

**Table S1.3**

Descriptive statistics of the frequency of scars (removals) on the dorsal and ventral faces of large cutting tool assemblages.

| Stat. | Crude LCTs/Handaxes | | | | Picks | | | | Large scrapers | |
| --- | --- | --- | --- | --- | --- | --- | --- | --- | --- | --- |
|  | ***MW2-L3*** | | ***MW2-L1&L2*** | | ***MW2-L3*** | | ***MW2-L1&L2*** | | ***MW2-L3*** | |
|  | *Dor.* | *Ven* | *Dor.* | *Ven.* | *Dor.* | *Ven.* | *Dor.* | *Ven.* | *Dor.* | *Ven.* |
| *n* | 4 | 4 | 29 | 29 | 2 | 2 | 6 | 6 | 6 | 6 |
| *Mean* | 8.3 | 4.3 | 9.0 | 5.7 | 6 | 5 | 10.8 | 5.2 | 3.7 | 0.8 |
| *S.D.* | 3.4 | 1.5 | 4.4 | 3.2 | 2.8 | 1.4 | 2.4 | 3.1 | 2.1 | 0.9 |
| *Min* | 5 | 3 | 2 | 0 | 4 | 4 | 7 | 0 | 1 | 0 |
| *Max* | 13 | 6 | 19 | 12 | 8 | 6 | 13 | 8 | 6 | 2 |

Abbreviations: Dor. = Dorsal face; Ven. = Ventral face.
