# Supplementary material for: Continuity and change in lithic techno-economy of the early Acheulian on the Ethiopian highland: A case study from locality MW2; the Melka Wakena site-complex
Source: PLoS One. 2022 Dec 7;17(12):e0277029. doi: 10.1371/journal.pone.0277029 (PMC9728887; doi:10.1371/journal.pone.0277029)
Supplement: S4 Table — (DOCX) [file pone.0277029.s013.docx]

**Table S1.4**

Descriptive statistics of core and hammerstone weights.

| Stat. | *MW2-L3* | | | | | *MW2-L1&L2* | | | | |
| --- | --- | --- | --- | --- | --- | --- | --- | --- | --- | --- |
|  | ***Hammerstones*** | | | | ***Cores*** | ***Hammerstones*** | | | | ***Cores*** |
|  | ***L*** | ***W*** | ***Th*** | ***Wei.*** | ***Wei.*** | ***L*** | ***W*** | ***Th*** | ***Wei.*** | ***Wei.*** |
| *n* | 46 | 46 | 46 | 46 | 259 | 6 | 6 | 6 | 6 | 40 |
| *Mean* | 98.9 | 82.1 | 70.7 | 798.5 | 586.4 | 85.5 | 76.1 | 64.1 | 505.6 | 718.4 |
| *S.D.* | 20.7 | 20.4 | 16.6 | 516.6 | 823.4 | 8.5 | 12.5 | 7.9 | 116.0 | 670.3 |
| *Min* | 57 | 48.4 | 45.3 | 173.4 | 8.1 | 76.9 | 62.3 | 52.9 | 376.7 | 20 |
| *Max* | 143.1 | 131.8 | 111.7 | 2185 | 7945 | 100.4 | 89.2 | 72.8 | 715.0 | 3150 |
