# Supplementary material for: Continuity and change in lithic techno-economy of the early Acheulian on the Ethiopian highland: A case study from locality MW2; the Melka Wakena site-complex
Source: PLoS One. 2022 Dec 7;17(12):e0277029. doi: 10.1371/journal.pone.0277029 (PMC9728887; doi:10.1371/journal.pone.0277029)
Supplement: S5 Table — (DOCX) [file pone.0277029.s014.docx]

**Table S1.5**

Physical properties of core assemblages from MW2 occupation layers.

| Attributes | *MW2-L3* | | | | | | *MW2-L1&L2* | | | |
| --- | --- | --- | --- | --- | --- | --- | --- | --- | --- | --- |
|  | *Glassy ign.* | *Ignimbrite* | *Pumice. ign.* | *Basalt* | *Scoria* | ***Total*** | *Glassy ign.* | *Ignimbrite* | *Basalt* | *Total* |
| State of preservation | | | | | | | | | | |
| Fresh | **50** (61.7) | **31** (24.2) | **2** (6.7) | **7** (50.0) | **1** (25.0) | **91** (35.4) | **16** (72.7) | **4** (28.6) | **2** (50.0) | **22** (55.0) |
| Slightly abraded | **24** (29.7) | **60** (46.9) | **13** (43.3) | **6** (42.9) | **3** (75.0) | **106** (41.2) | **5** (22.7) | **3** (21.4) | **1** (25.0) | **9** (22.5) |
| Abraded | **7** (8.6) | **33** (25.8) | **14** (46.7) | **1** (7.1) | **-** | **55** (21.4) | **1** (4.5) | **6** (42.9) | **1** (25.0) | **8** (20.0) |
| Heavily abraded | **-** | **4** (3.1) | **1** (3.3) | **-** | **-** | **5** (1.9) | **-** | **1** (7.1) | **-** | **1** (2.5) |
| Patination |  | | | | | | | | | |
| No patina | **72** (88.9) | **123** (96.1) | **29** (96.7) | **9** (64.3) | **2** (50.0) | **235** (91.4) | **8** (36.4) | **5** (35.7) | **1** (25.0) | **14** (35.0) |
| Patinated | **9** (11.1) | **5** (3.9) | **1** (3.3) | **5** (35.7) | **2** (50.0) | **22** (8.6) | **14**(63.6) | **9** (64.3) | **3** (75.0) | **26** (65.0) |
| Concretion |  | | | | | | | | | |
| No concretion | **4** (5.0) | **22** (17.2) | **5** (16.7) | **-** | **-** | **31** (12.1) | **12** (54.5) | **7** (50.0) | **1** (25.0) | **20** (50.0) |
| Sand | **74** (91.3) | **105** (82.0) | **25** (83.3) | **10** (71.4) | **2** (50.0) | **216** (84.0) | **8** (36.4) | **2** (14.3) | **3** (75.0) | **13** (32.5) |
| Sand & Conglomerate | **1** (1.2) | **1** (0.8) | **-** | **3** (21.4) | **1** (25.0) | **6** (2.3) | **1** (4.5) | **1** (7.1) | **-** | **2** (5.0) |
| Carbonate | **-** | **-** | **-** | **-** | **-** | **-** | **1** (4.5) | **2** (14.3) | **-** | **3** (7.5) |
| Carbonate & sand | **2** (2.5) | **-** | **-** | **1** (7.1) | **1** (25.0) | **4** (1.6) | **-** | **2** (14.3) | **-** | **2** (5.0) |
| Cortex |  | | | | | | | | | |
| Non-cortical | **50** (61.7) | **24** (18.7) | **1** (3.3) | **3** (21.4) | **-** | **78** (30.4) | **10** (45.5) | **2** (14.3) | **2** (50.0) | **14** (35.0) |
| 0–25% | **21** (26.0) | **39** (30.5) | **5** (16.7) | **4** (28.6) | **2** (50.0) | **71** (27.6) | **10** (45.5) | **2** (14.3) | **1** (25.0) | **13** (32.5) |
| 26–50% | **3** (3.7) | **27** (21.1) | **7** (23.3) | **1** (7.1) | **1** (25.0) | **39** (15.2) | **1** (4.5) | **3** (21.4) | **-** | **4** (10.0) |
| 51–75% | **6** (7.4) | **31** (24.2) | **13** (43.3) | **4** (28.6) | **1** (25.0) | **55** (21.4) | **1** (4.5) | **5** (35.7) | **1** (25.0) | **7** (17.5) |
| 76–100% | **1** (1.2) | **7** (5.5) | **4** (13.3) | **2** (14.2) | **-** | **14** (5.4) | **-** | **2** (13.3) | **-** | **2** (5.0) |
| *Total* | **81** | **128** | **30** | **14** | **4** | **257** | **22** | **14** | **4** | **40** |
