# Supplementary material for: Continuity and change in lithic techno-economy of the early Acheulian on the Ethiopian highland: A case study from locality MW2; the Melka Wakena site-complex
Source: PLoS One. 2022 Dec 7;17(12):e0277029. doi: 10.1371/journal.pone.0277029 (PMC9728887; doi:10.1371/journal.pone.0277029)
Supplement: S6 Table — (DOCX) [file pone.0277029.s015.docx]

**Table S1.6**

Physical properties of LCTs from MW2 occupation layers.

| Attributes | *Picks* | | *Handaxes* | | *Cleavers* |
| --- | --- | --- | --- | --- | --- |
|  | *MW2-L3* | *MW2-L1&L2* | *MW2-L3* | *MW2-L1&L2* | *MW2-L1&L2* |
| *State of preservation* | | | | | |
| Fresh | **1**(50%) | **6**(100%) | **3**(75%) | **29**(100%) | **1**(100%) |
| Slightly abraded | **1**(50%) | **-** | **1**(25%) | - | - |
| *Patination* | | | | | |
| No patina | **2**(100%) | **2**(33.3%) | **4**(100%) | **16**(55.2%) | **1**(100%) |
| Patinated | - | **4**(66.7%) | - | **13**(44.8%) | - |
| *Concretion* | | | | | |
| No concretion | - | **5**(83.3%) | - | **19**(65.5%) | **1**(100%) |
| Sand | **2**(100%) | - | **4**(100%) | **4**(13.8%) | - |
| Carbonate | - | **1**(16.7%) |  | **6**(20.7%) | - |
| *Cortex* | | | | | |
| Non-cortical | **1**(50%) | **4**(66.7%) | **2**(50%) | **18**(62.1%) | **1**(100%) |
| 0–25% | **1**(50%) | **2**(33.3%) | **2**(50%) | **10**(34.5%) | **-** |
| 26–50% | **-** | - | - | **1**(3.4%) | **-** |
| 51–75% | **-** | - | - | **-** | **-** |
| 76–100% | **-** | - | - | **-** | **-** |
| *Total* | **2** | **6** | **4** | **29** | **1** |
